# Supplementary material for: The eco-evolutionary assembly of complex communities with multiple interaction types
Source: Nat Commun. 2026 Mar 5;17:3511. doi: 10.1038/s41467-026-70117-8 (PMC13083869; doi:10.1038/s41467-026-70117-8)
Supplement: Supplementary file 1 — Supplementary Information [file 41467_2026_70117_MOESM1_ESM.pdf]

Supplementary material for the paper '*The eco-evolutionary assembly of complex communities with multiple interaction types*'

Gui Araujo[1] & Miguel Lurgi[1,*]

[1]Department of Biosciences, Swansea University. Swansea, SA2 8PP. UK.

* corresponding author: miguel.lurgi@swansea.ac.uk

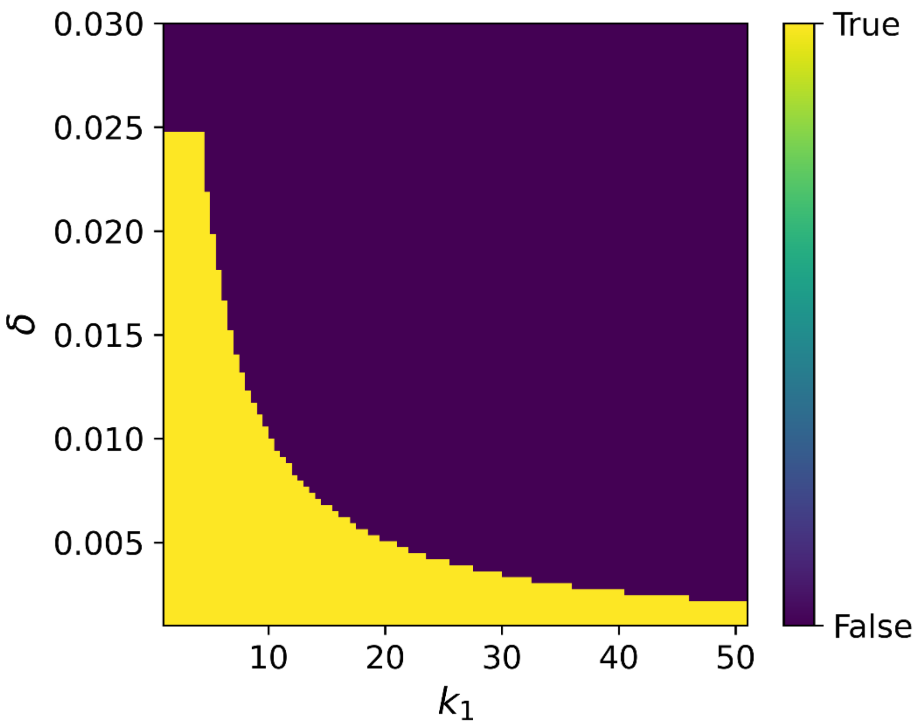

Figure S1. **Threshold in the mean-field approximation of the model.** Value of the inequality $\frac{\mu x}{1+h_m k_1 \mu x} - \delta > 0$ that represents the positive net effect of mutualism necessary for Type 2 communities. In the region where the inequality is true (yellow), it is beneficial for a species to accumulate more mutualistic interactions. The inequality is shown in terms of the average number of mutualistic interactions per species (k_1) and the average cost in baseline reproduction per mutualistic interaction (δ). Parameter values: $\mathrm{r} = 0.1,\ \mu = 0.5,\ h_m = 0.1,\ k_2 = 2,\ \gamma = 1.$

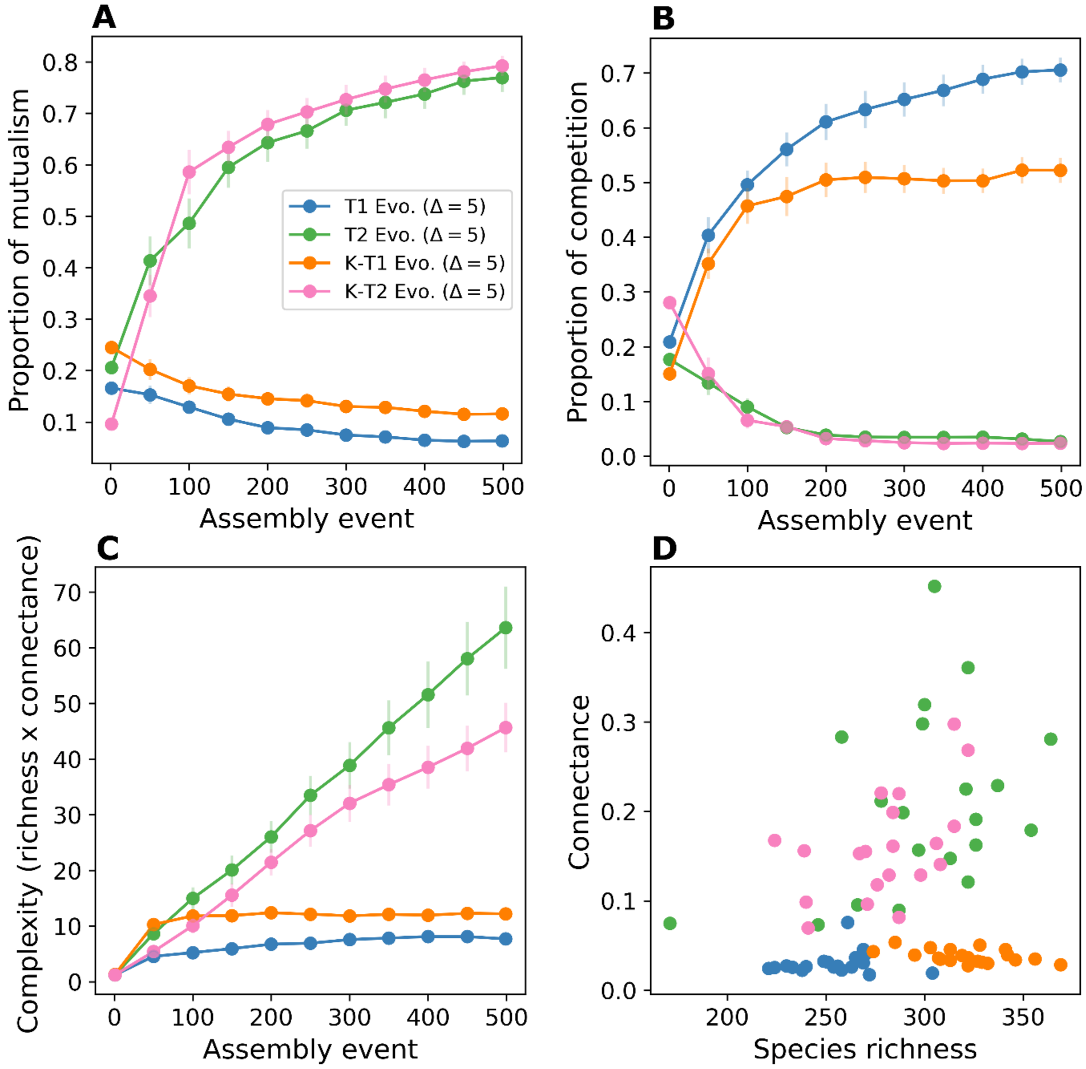

Figure S2. **The scale of intraspecific competition establishes the threshold of benefits of interactions.** The plots show the average values of n=20 samples (i.e. replicated simulations of the model) for every 50 assembly events (dots) and the vertical lines show the standard errors ($SD/\sqrt{n}$). We simulate the effect on community types of changing the mean of intraspecific competition, which rescales the benefits of interactions (i.e. interaction strength σ in relation to the cost of positive interactions δ) for assembly by evolution ($\Delta = 5$). T1 and T2 curves (blue and green) are Type 1 and 2 communities, respectively defined by ($\sigma = 0.05, \delta = 0.025$) and ($\sigma = 0.2, \delta = 0.01$), as in Figure 2. The K-T1 curve is originally defined as a T2, but it also has a greater average intraspecific competition, with $s_i \propto r_i/[log\mathcal{N}(-4{,}0.5^2)]$ instead of $s_i \propto r_i/[log\mathcal{N}(-2.2{,}0.5^2)]$. Increasing the intraspecific competition from T2 to K-T1 transforms the outcome into a type 1 community (green and orange curves). The same applies to T1 and K-T2, in which decreasing the intraspecific competition to $s_i \propto r_i/[log\mathcal{N}(-0.4{,}0.5^2)]$ transforms the outcome into a Type 2 community (blue and magenta curves). (A-B) The characteristic outcome of interaction-type composition of both community types flips by changing s_i. (C-D) Complexity and its components, richness and connectance, also flip accordingly. Parameter values are the same as in Figure 2.

Supplementary Note 1: Complementary simulations (Figs S3 to S22)

The panels from Figs S3 to S22 comprise additional simulations that explore the sensitivity of results to parameter changes or extend our core assumptions to adjacent interesting scenarios. Notable observations are discussed in the captions , which focus on detailing the goals and outcomes of the simulations. For all panels, we have: (A-F) Assembly histories shown in particular metrics as they change through 500 assembly events (proportion of mutualism, proportion of consumer-resource, proportion of competition, species richness, connectance, and complexity). Dots represent the mean of n=4 replicate simulations sampled every 50 events, with vertical lines indicating the standard error ($SD/\sqrt{n}$). In general, n=4 samples are enough to robustly confirm the trends. (G) Final values of network connectance versus species richness for the communities shown in panels A and C, measured at assembly event 500. Each dot represents a single simulation. (H-I) The same as in Fig S23 for the main text simulations, topological modularity and degree entropy increase at the end of simulations. Metrics were analysed as the relative increase from the average random network with the same complexity, calculated with n=50 samples. Values were subtracted by the random average and then divided by it, resulting in a relative increase and measuring how much is driven by the structure generated in the assembly process. Apart from the differences stated in the captions, all simulations were the same as the ones analysed in the main text, with the same definitions of scenarios and the same parameter values.

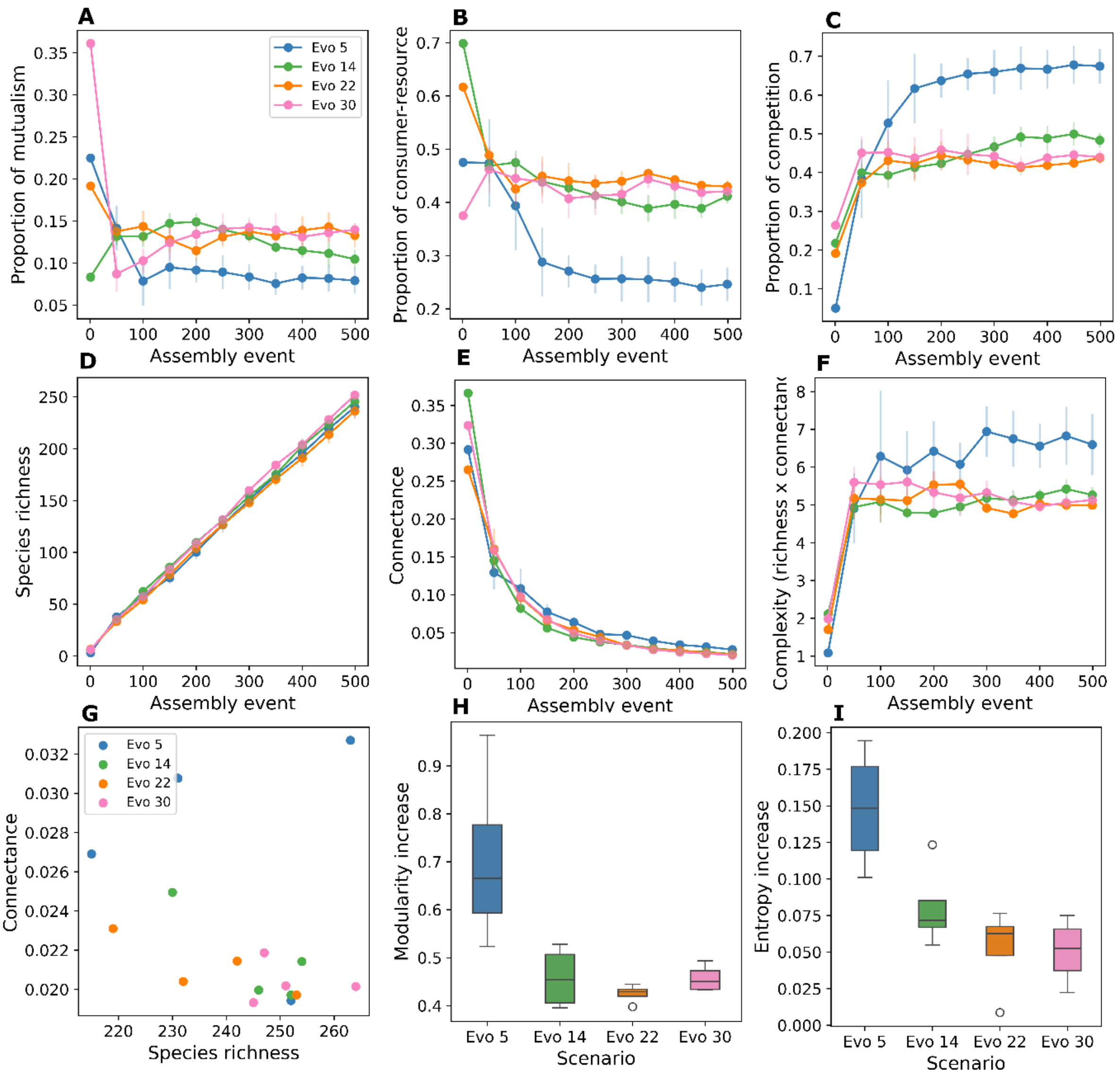

Figure S3. **Evolution with additional inheritance strength values (Δ), Type 1.** Evo 5 and Evo 30 are the same scenarios as in the main text. Here, we include two intermediate scenarios, which resemble Evo 30. This suggests a nonlinear relation with very high fidelity of inheritance (Evo 5) a distinct and more strict mode in Type 1 communities. This might stem from a more effective selection of competition. See more details about the panels in the text above.

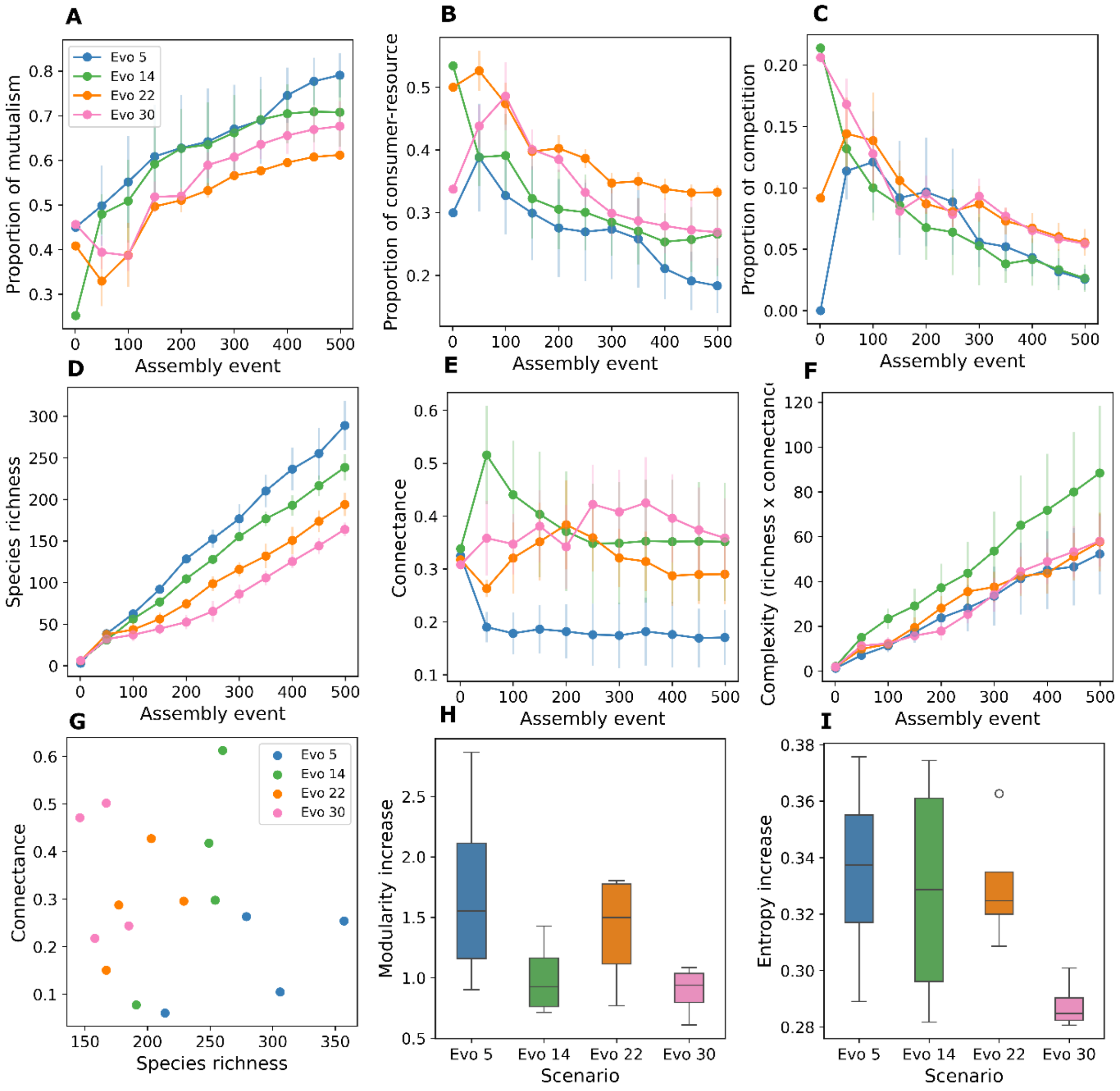

Figure S4. **Evolution with additional inheritance strength values (Δ), Type 2.** Same as S3, now for Type 2 communities. Here, we see a more linear relation and the gradual change in 'complexity mode' is visible: stronger inheritance prioritises species richness while weaker inheritance prioritises connectance. In (G) we can see the gradual shift while in (F) we see that complexity remains in the same range. See more details about the panels in the text above (text before Fig. S3).

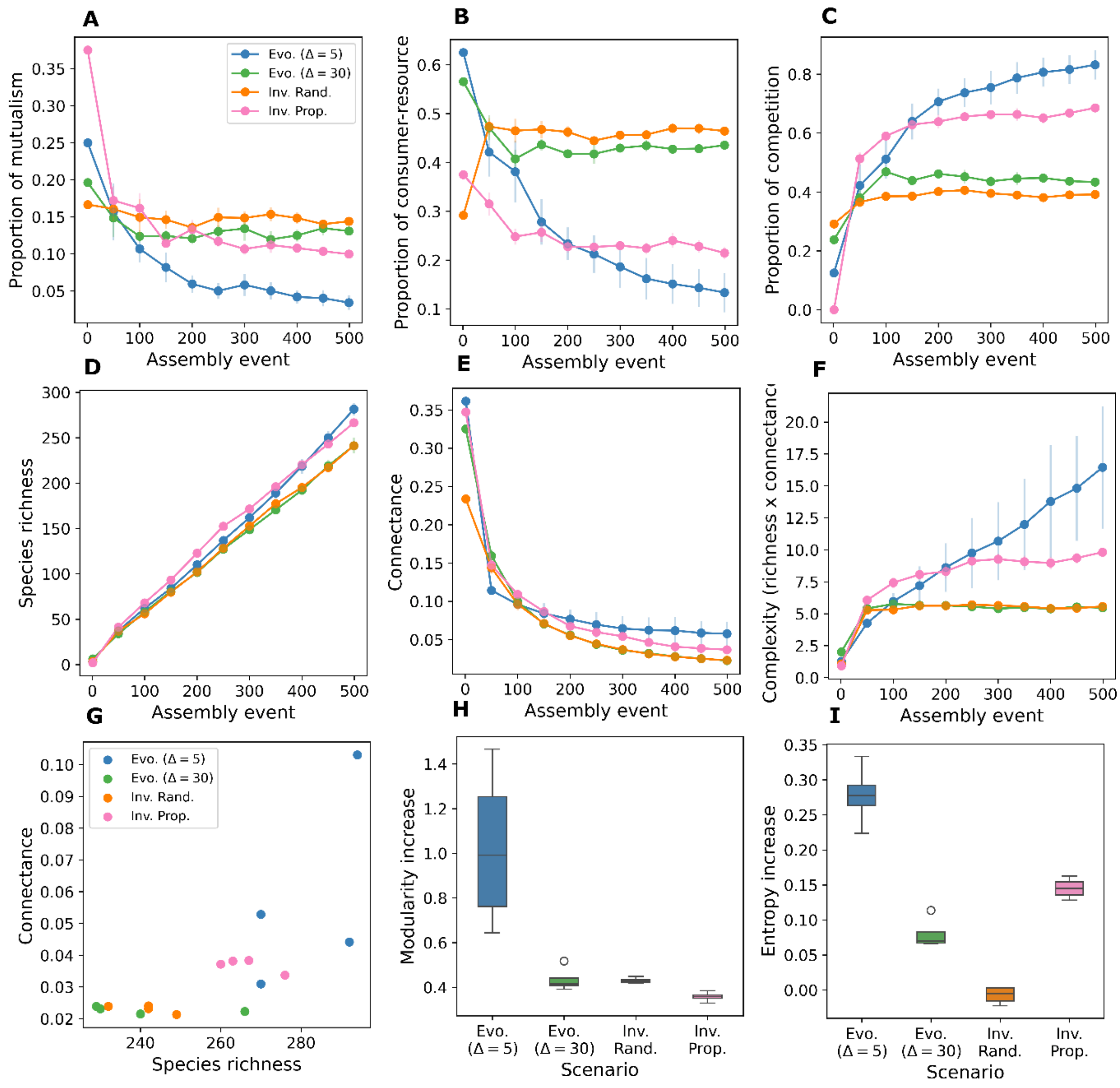

Figure S5. **Alternative T1 scenario with even lower interaction strength ($\sigma = 0.002$).** Further exploring the parameter range of Type 1 communities, we see that an interaction strength five times smaller yields generally equivalent results. See more details about the panels in the text above (text before Fig. S3).

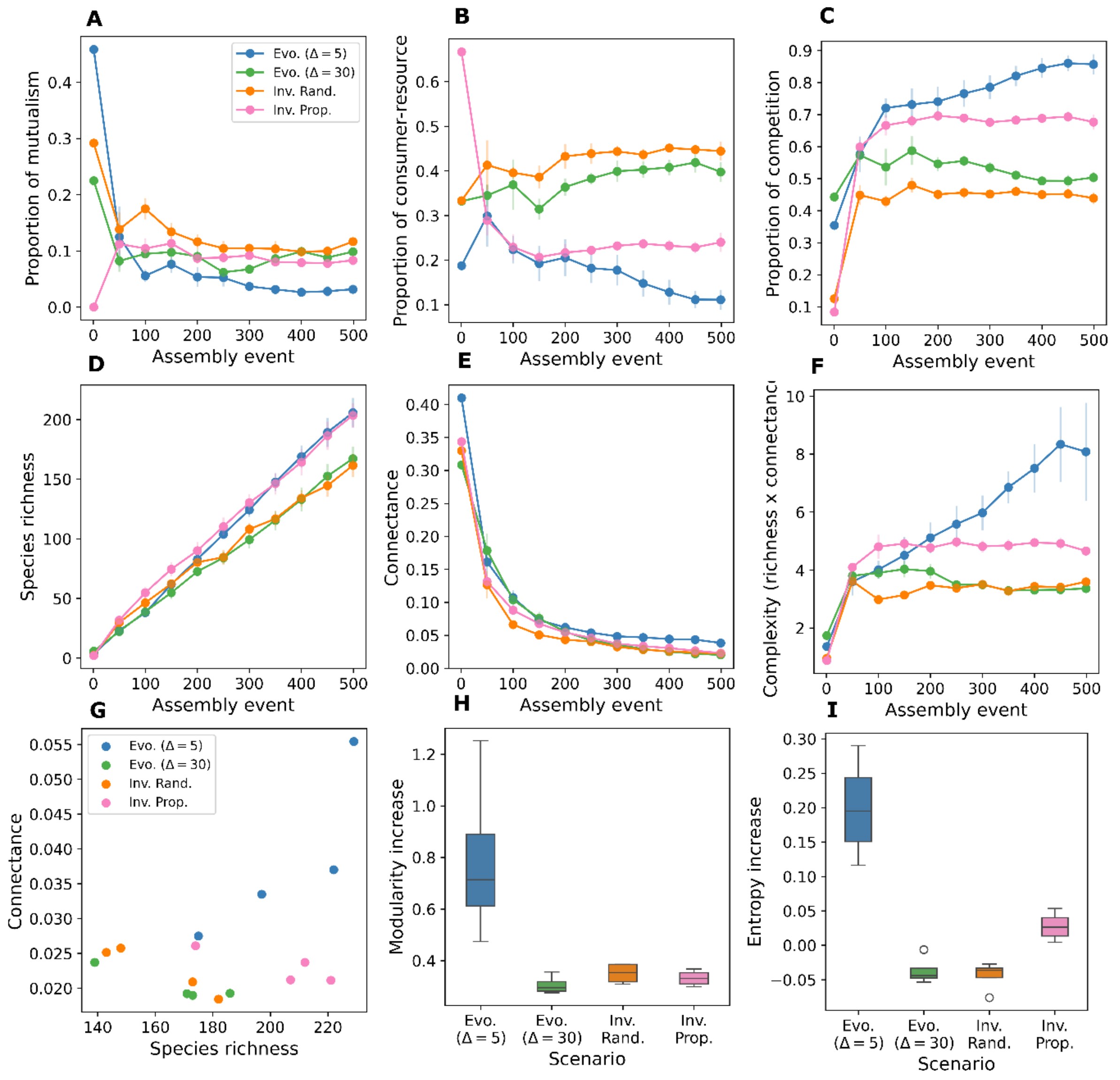

Figure S6. **Alternative T1 scenario with even higher cost of positive interactions ($\delta = 0.04$).** Further exploring the parameter range of Type 1 communities, we see that a 60% higher harvesting cost yields generally equivalent results. See more details about the panels in the text above (text before Fig. S3).

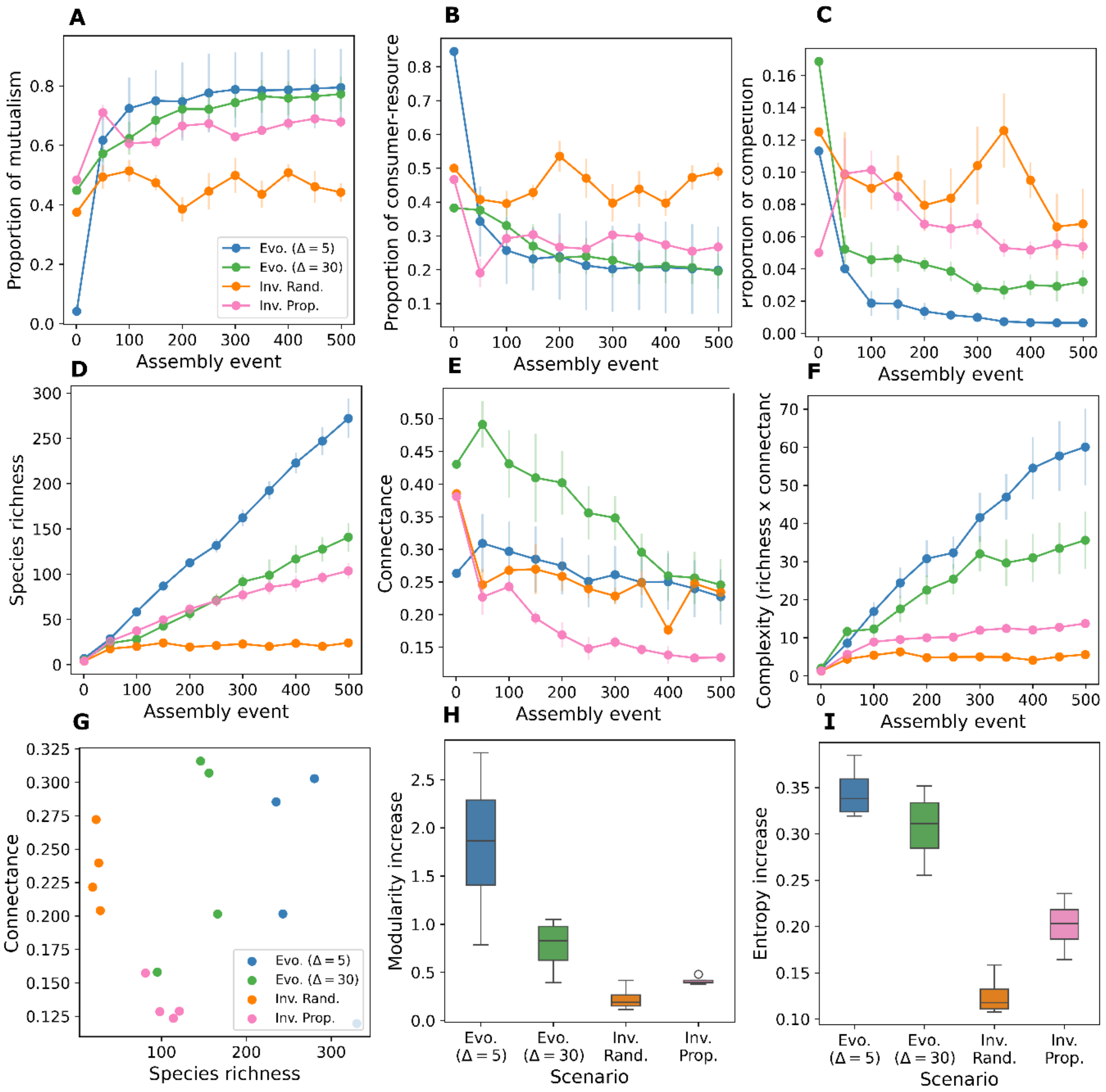

Figure S7. **Alternative T2 scenario with even higher interaction strength ($\sigma = 0.5$).** Further exploring the parameter range of Type 2 communities, we see that an interaction strength 2.5 times higher yields generally equivalent results. See more details about the panels in the text above (text before Fig. S3).

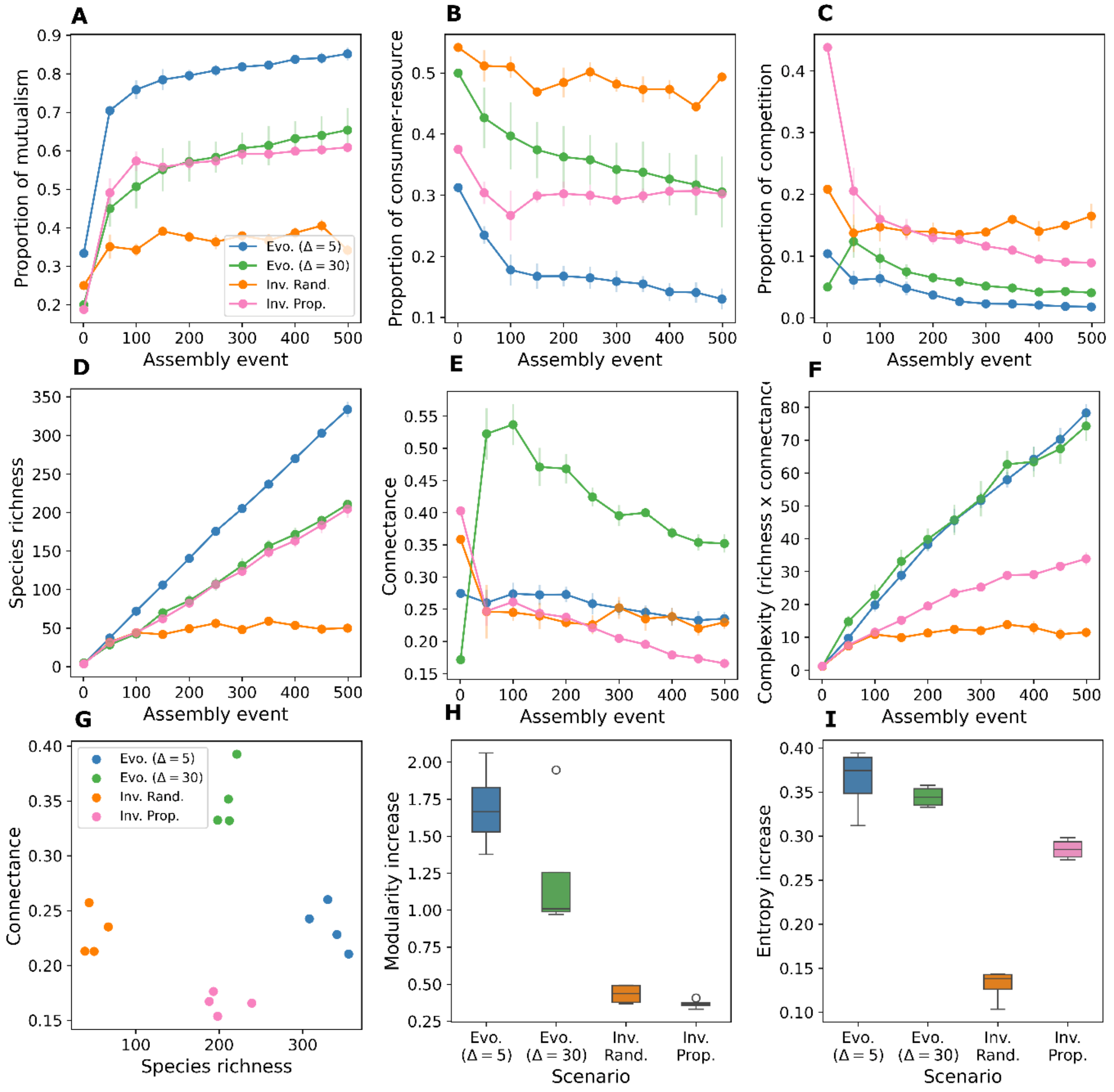

Figure S8. **Alternative T2 scenario with even lower cost of positive interactions ($\delta = 0.001$).** Further exploring the parameter range of Type 2 communities, we see that a 10 times lower harvesting cost yields generally equivalent results. See more details about the panels in the text above (text before Fig. S3).

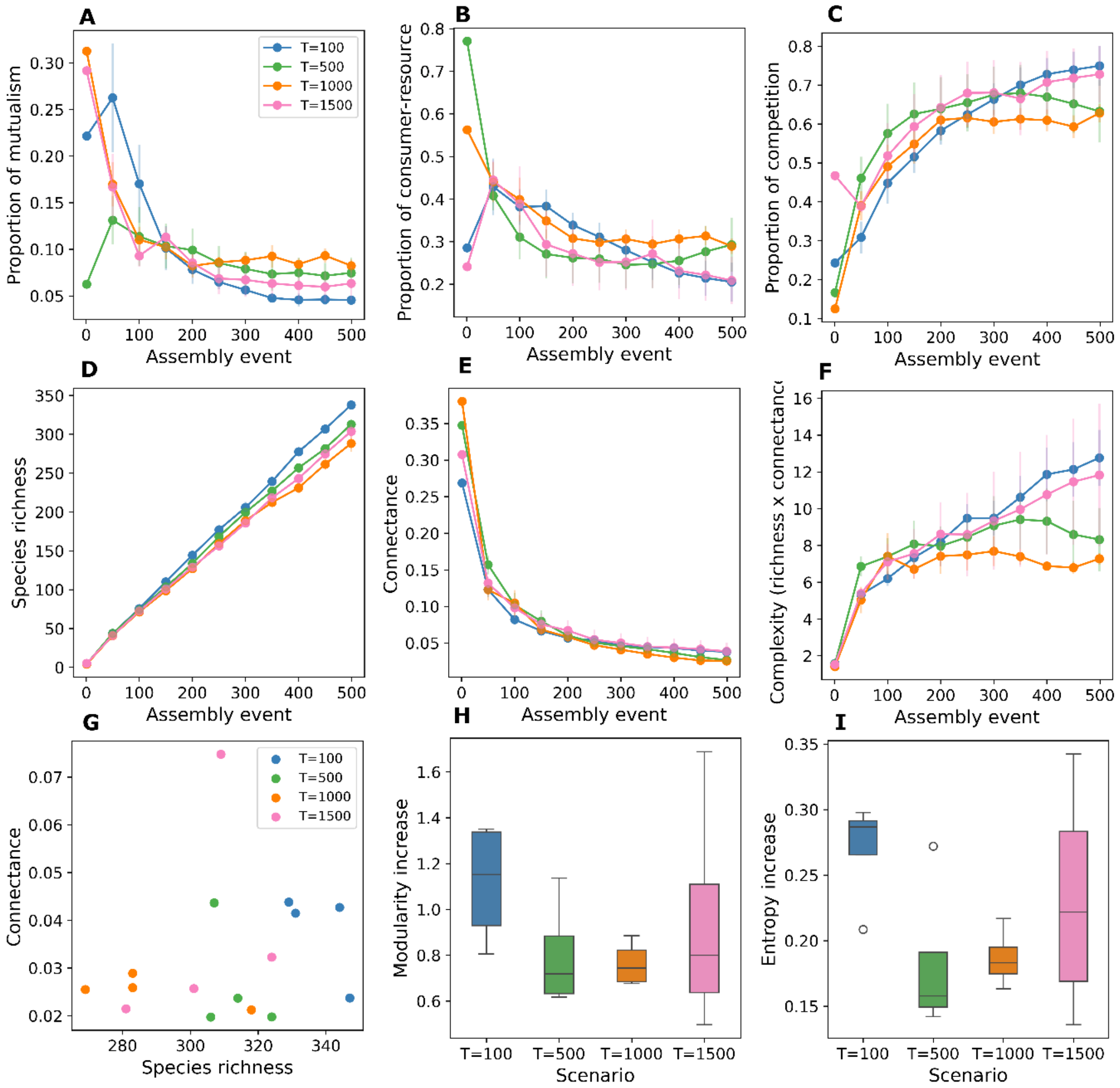

Figure S9. **Type 1 Evolution ($\Delta = 5$) with faster, out-of-equilibrium assembly events.** Generally, equilibrium was attained at around T=2000 in simulations. Here, we simulate faster Evo 5 assembly for Type 1 communities and results are generally the same for all scenarios. See more details about the panels in the text above (text before Fig. S3).

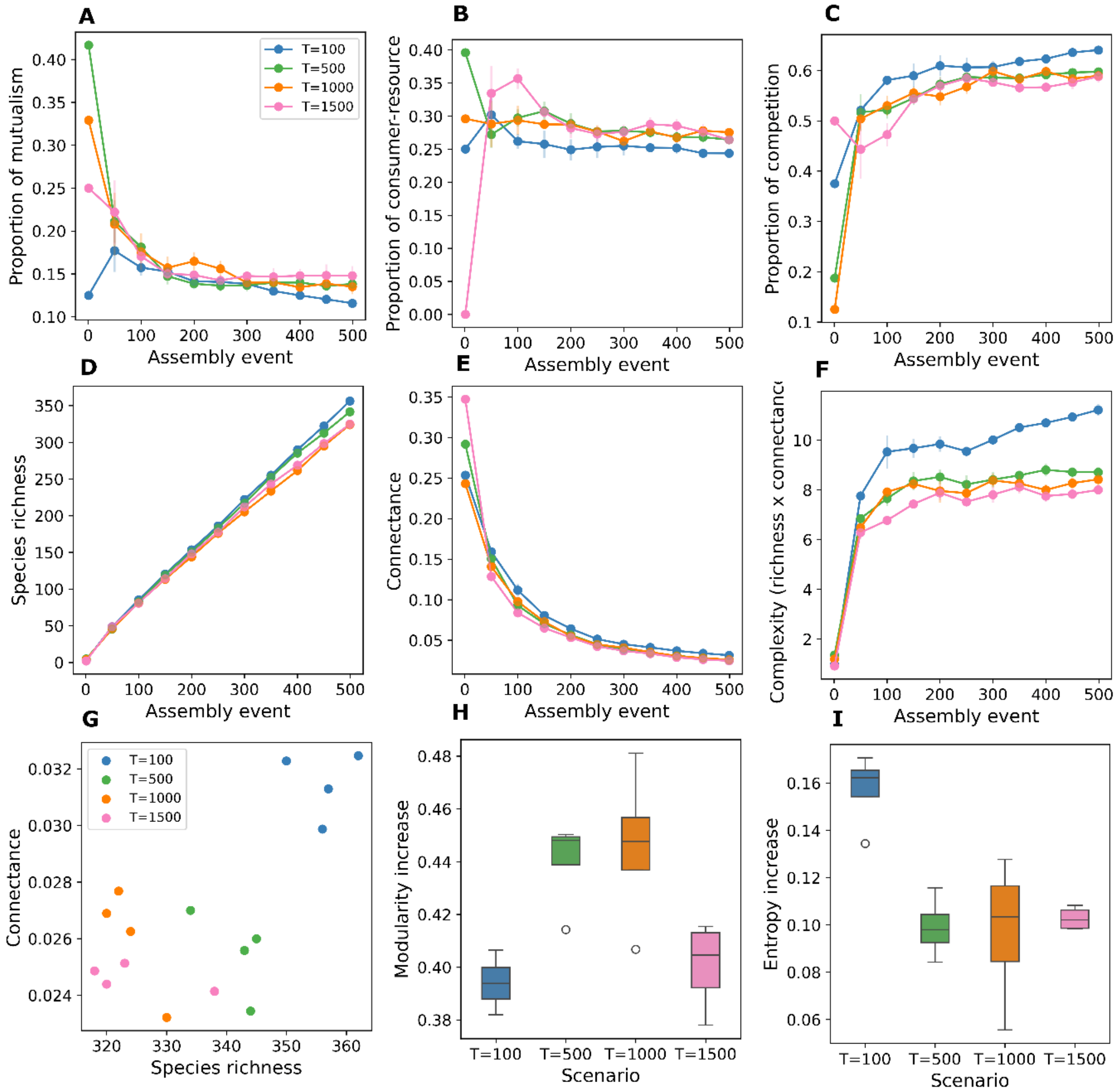

Figure S10. **Type 1 Invasion Prop. with faster, out-of-equilibrium assembly events.** Same as above, now for Inv. Prop., and only with extremely fast assembly (~20 times faster) there is a slight change in results: connectance and species richness are slightly higher, causing complexity to be higher. See more details about the panels in the text above (text before Fig. S3).

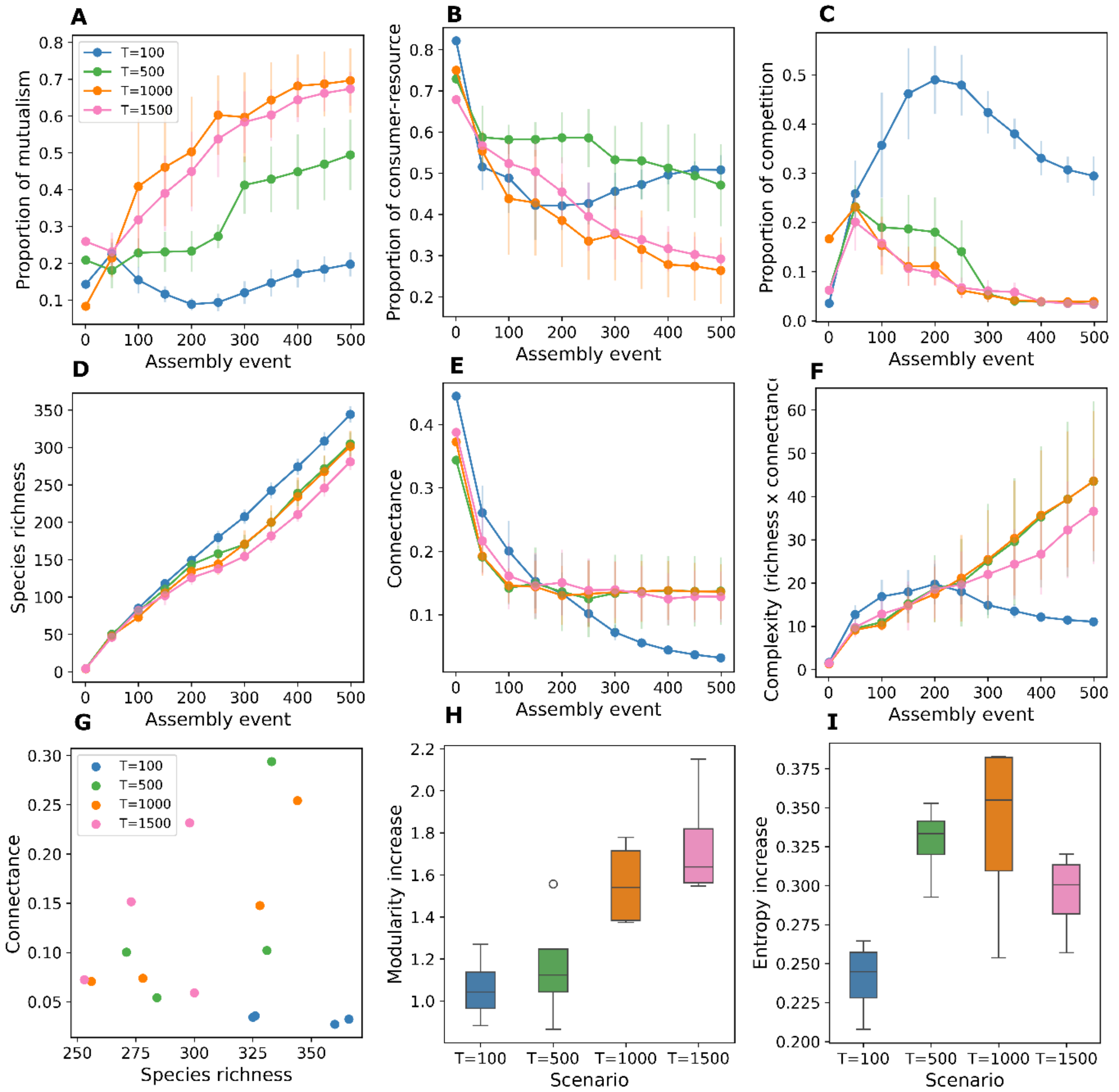

Figure S11. **Type 2 Evolution (Δ = 5) with faster, out-of-equilibrium assembly events.** Same as above, now for Evo 5 Type 2 scenario. For extremely fast assembly (~20 times faster), simulations behave closer to Type 1 communities. This might be related to lack of time for selection to be effective. The results seem to indicate that this scenario is just slower to accrue the selected properties. See more details about the panels in the text above (text before Fig. S3).

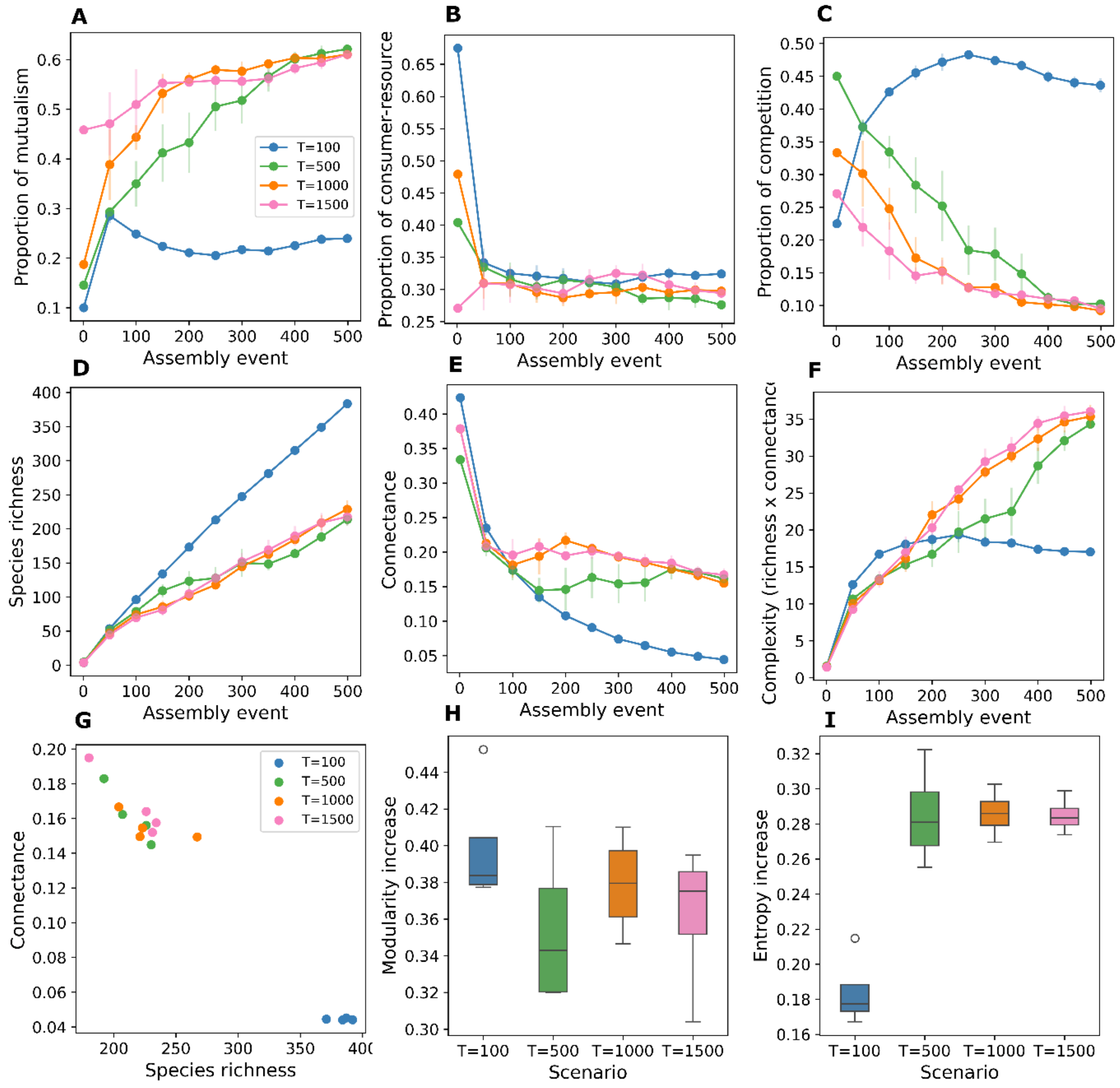

Figure S12. **Type 2 Invasion Prop. with faster, out-of-equilibrium assembly events.** Same as above, now for Inv. Prop. Type 2 scenario. The analysis is the same as above, all scenarios are equivalent except for extremely fast assembly. See more details about the panels in the text above (text before Fig. S3).

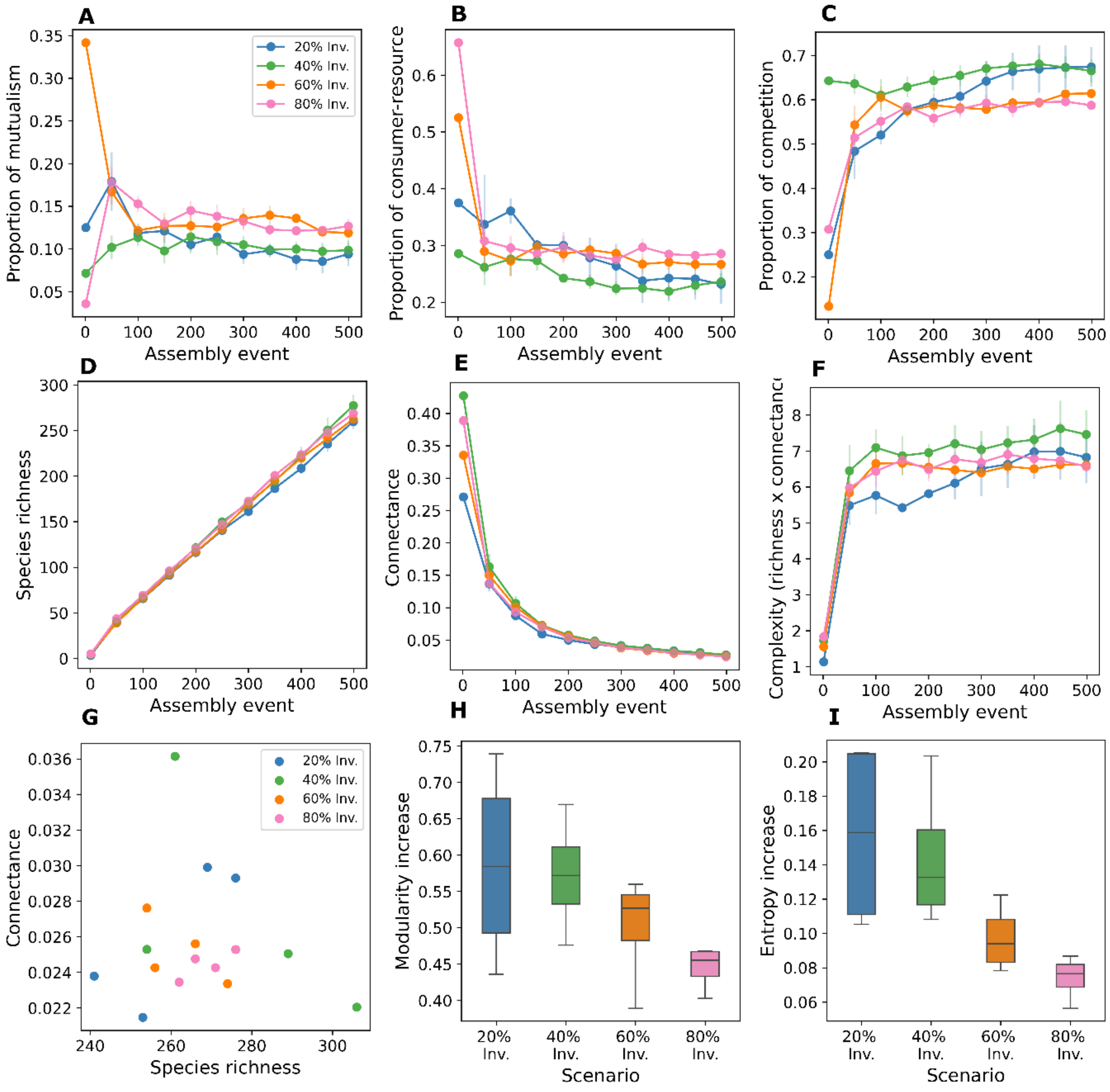

Figure S13. **Type 1 mixed assembly with different proportions of Evolution ($\Delta = 5$) and Invasion Prop.** These scenarios mix Evo 5 and Inv. Prop. assembly in Type 1 communities, going from 20% to 80% of invasion. Since these two scenarios are very similar in Type 1 communities, results are as expected. See more details about the panels in the text above (text before Fig. S3).

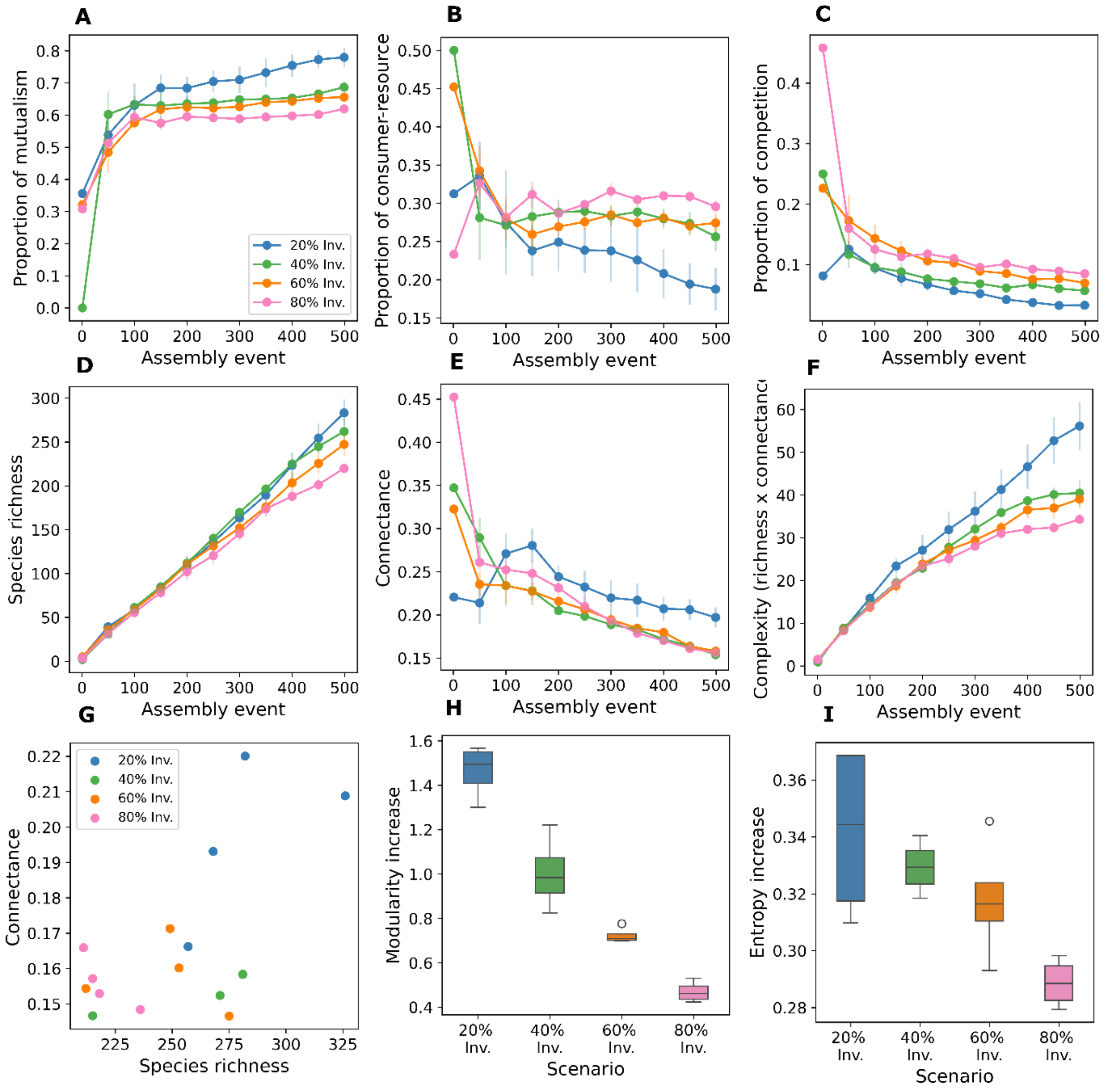

Figure S14. **Type 2 mixed assembly with different proportions of Evolution ($\Delta = 5$) and Invasion Prop.** These scenarios mix Evo 5 and Inv. Prop. assembly in Type 2 communities, going from 20% to 80% of invasion. In this case, we see a generally linear transition between Evo 5 and Inv Prop, but close to pure evolution there is a nonlinearity, suggesting an enhanced potential of selection by pure inheritance. See more details about the panels in the text above (text before Fig. S3).

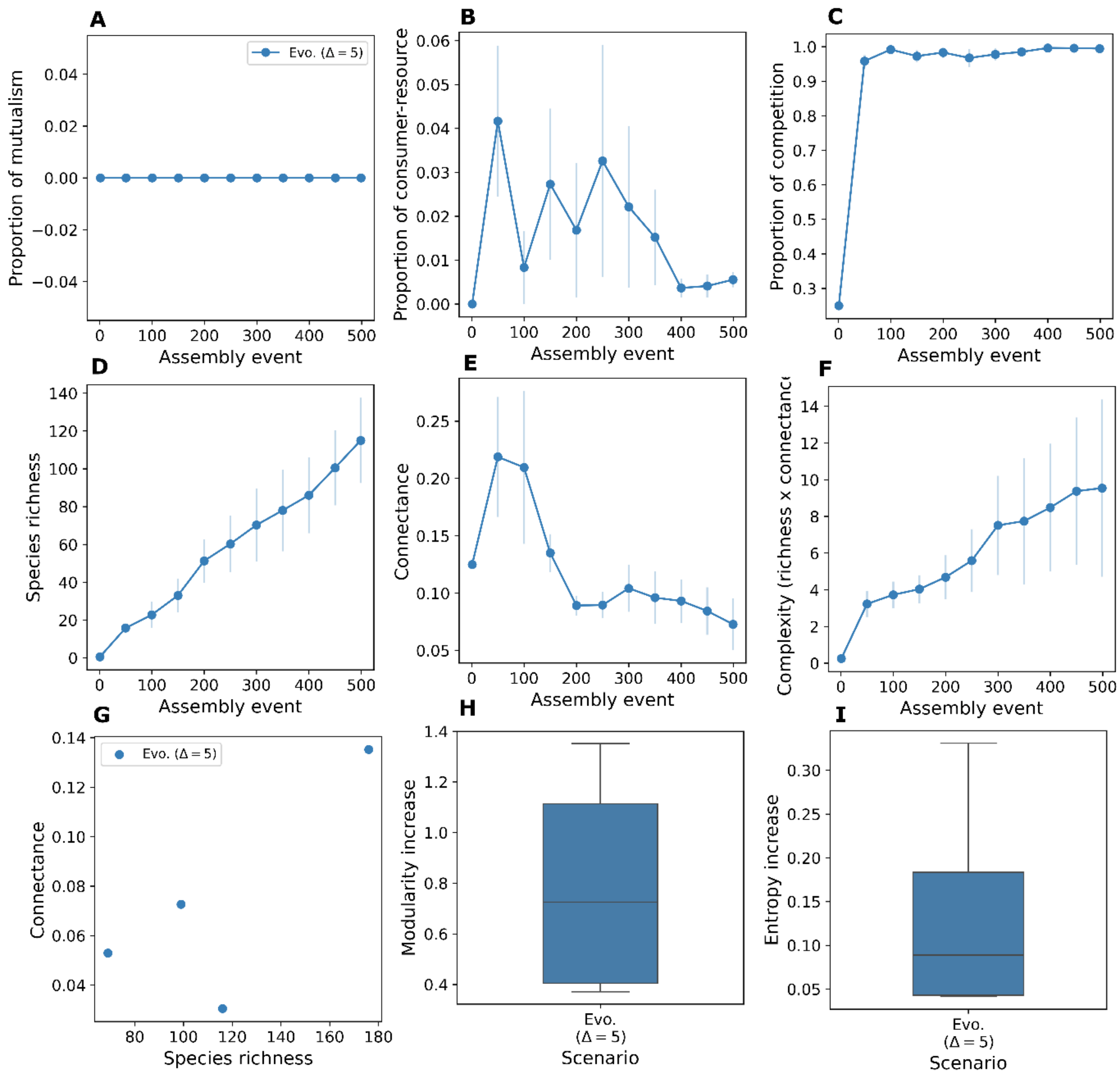

Figure S15. **Type 1 scenarios with lower intrinsic growth rate (mean $r = 0.02$).** Simulations with five times lower average growth rate, for Type 1 communities. Only Evo 5 scenarios were able to sustain a community, and mutualistic interactions were completely unsustainable. This suggests that strong inheritance can build a community even in face of very harsh conditions of survival and reproduction. See more details about the panels in the text above (text before Fig. S3).

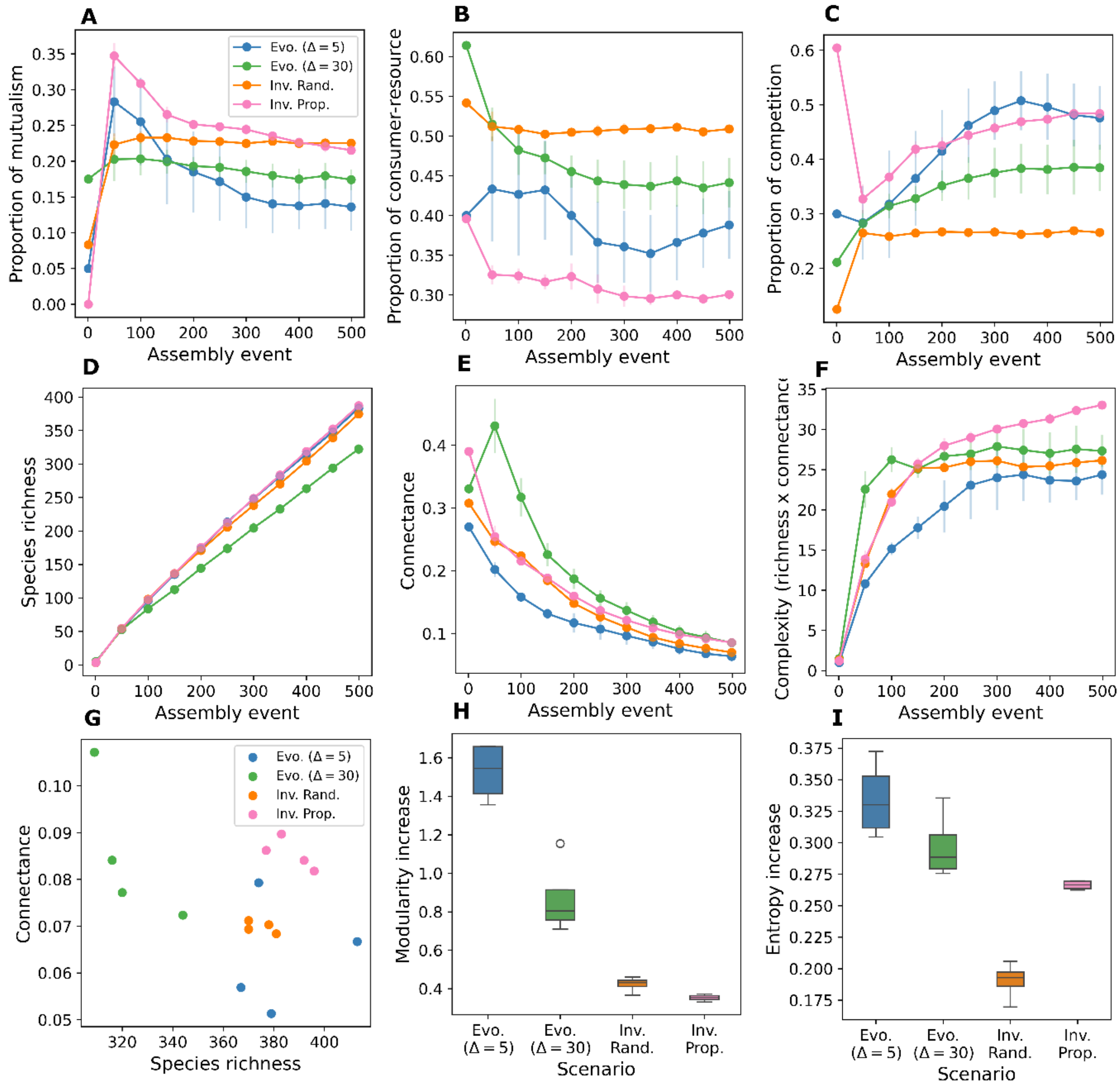

Figure S16. **Type 1 scenarios with higher intrinsic growth rate (mean $r = 0.5$).** Simulations with five times higher average growth rate, for Type 1 communities. Here, selection was largely relaxed, with much fewer overall extinctions. This resulted in more balanced interaction types and higher complexity. See more details about the panels in the text above (text before Fig. S3).

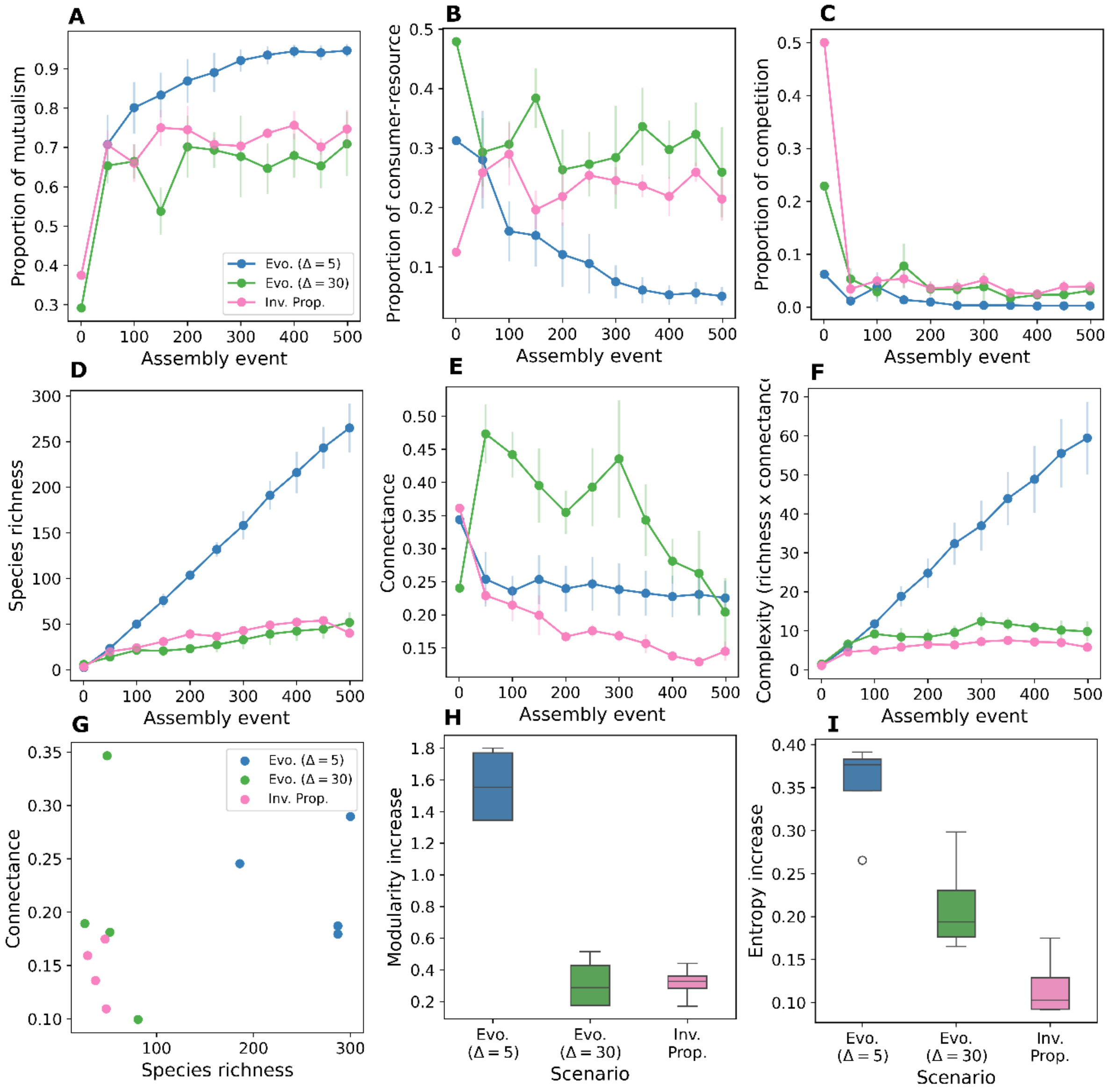

Figure S17. **Type 2 scenarios with lower intrinsic growth rate (mean $r = 0.02$).** Simulations with five times lower average growth rate, for Type 2 communities. Here, Inv Rand simulations could not sustain themselves, but Evo 30 and Inv Prop could barely grow. As in Type 1, only Evo 5 simulations could normally grow in this condition. See more details about the panels in the text above (text before Fig. S3).

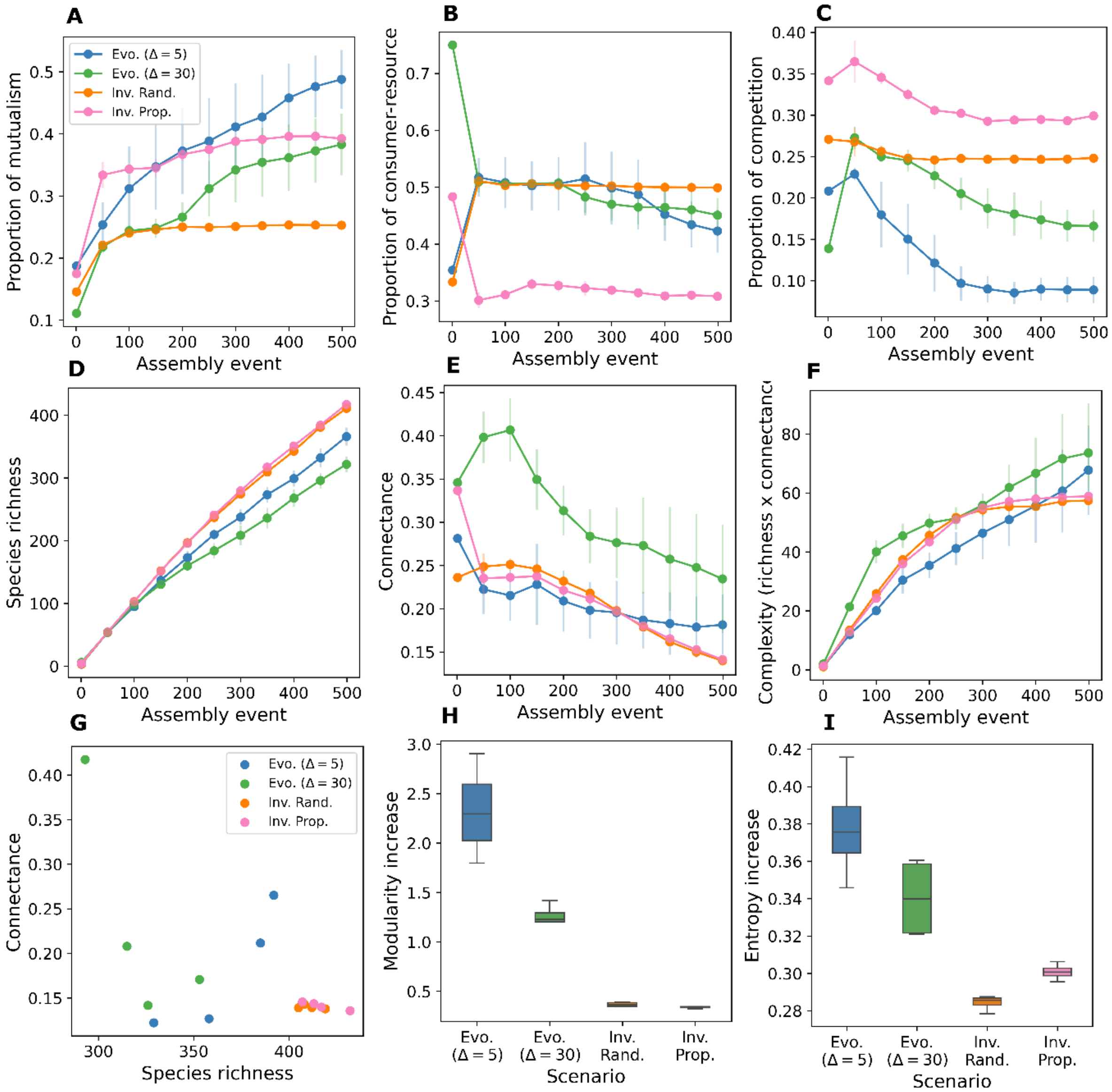

Figure S18. **Type 2 scenarios with higher intrinsic growth rate (mean $r = 0.5$).** Simulations with five times higher average growth rate, for Type 2 communities. As with Type 1, extinctions were generally fewer and selection was weakened. See more details about the panels in the text above (text before Fig. S3).

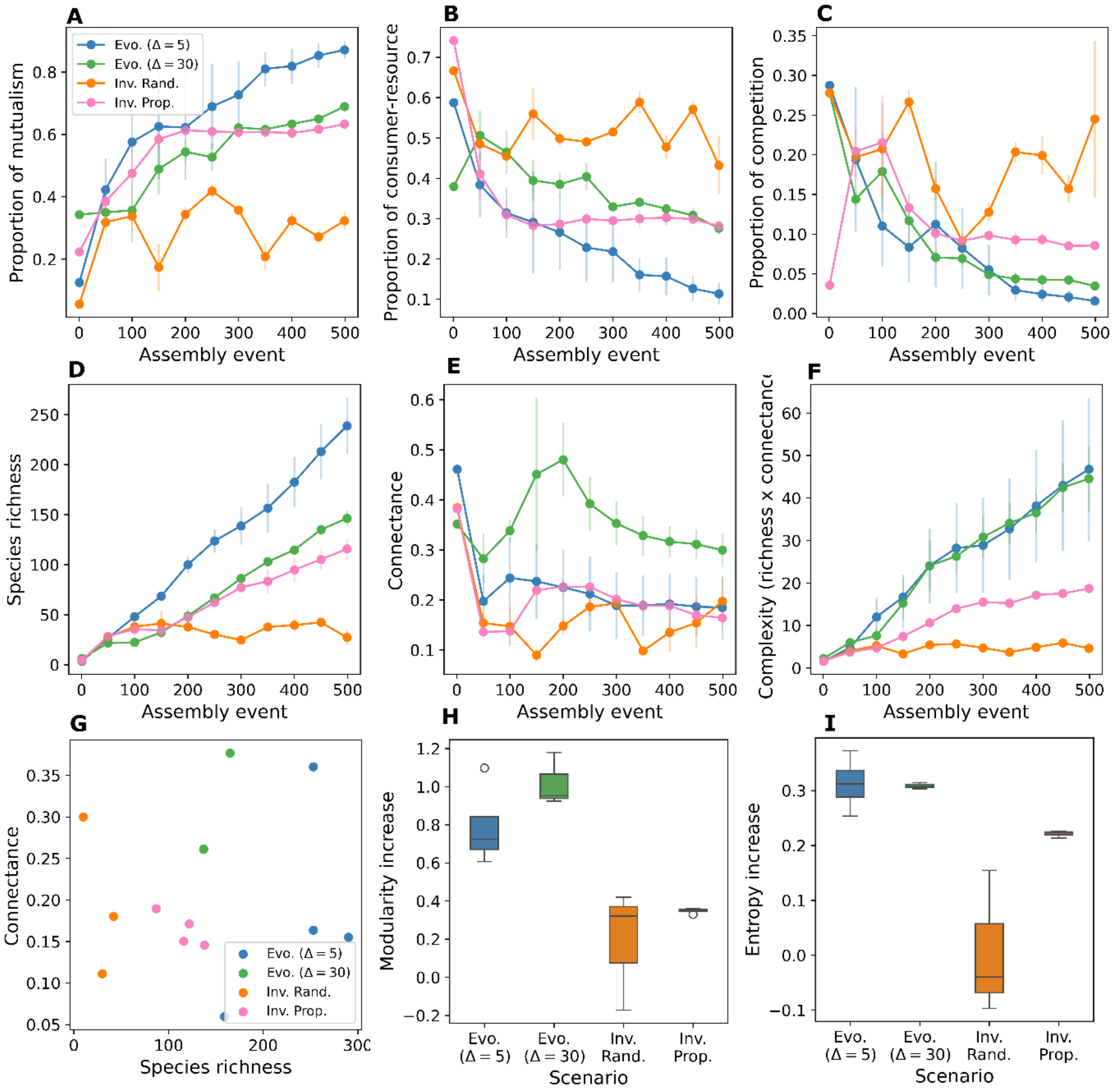

Figure S19. **Type 1 scenarios with lower intraspecific competition (mean of lognormal is -0.4 instead of -2.2).** As seen in Fig. S2, lowering the intraspecific competition in Type 1 communities shifts the behaviour to Type 2 communities. See more details about the panels in the text above (text before Fig. S3).

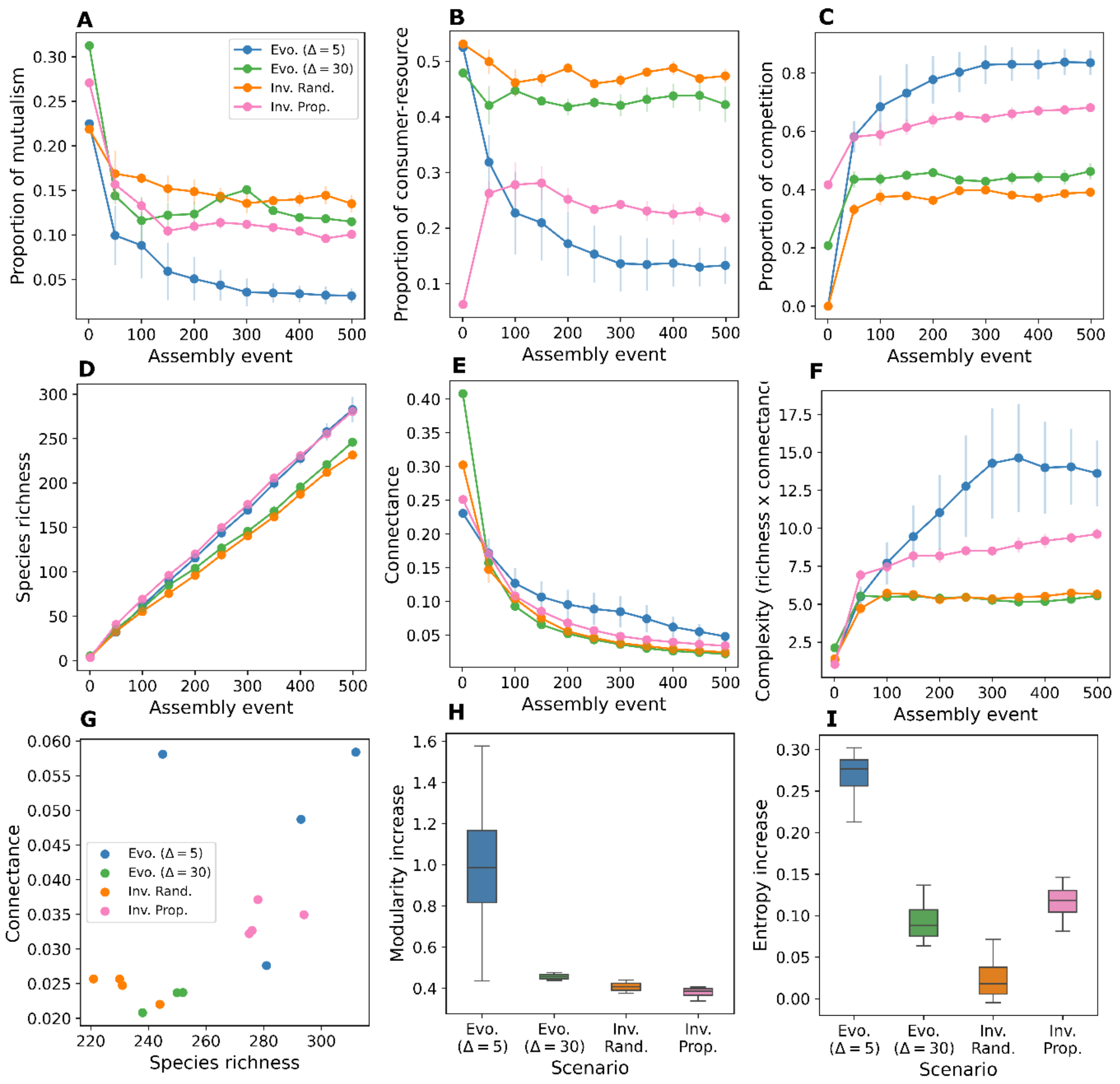

Figure S20. **Type 1 scenarios with higher intraspecific competition (mean of lognormal is -4 instead of -2.2).** Increasing the intraspecific competition in Type 1 communities generally does not change results. See more details about the panels in the text above (text before Fig. S3).

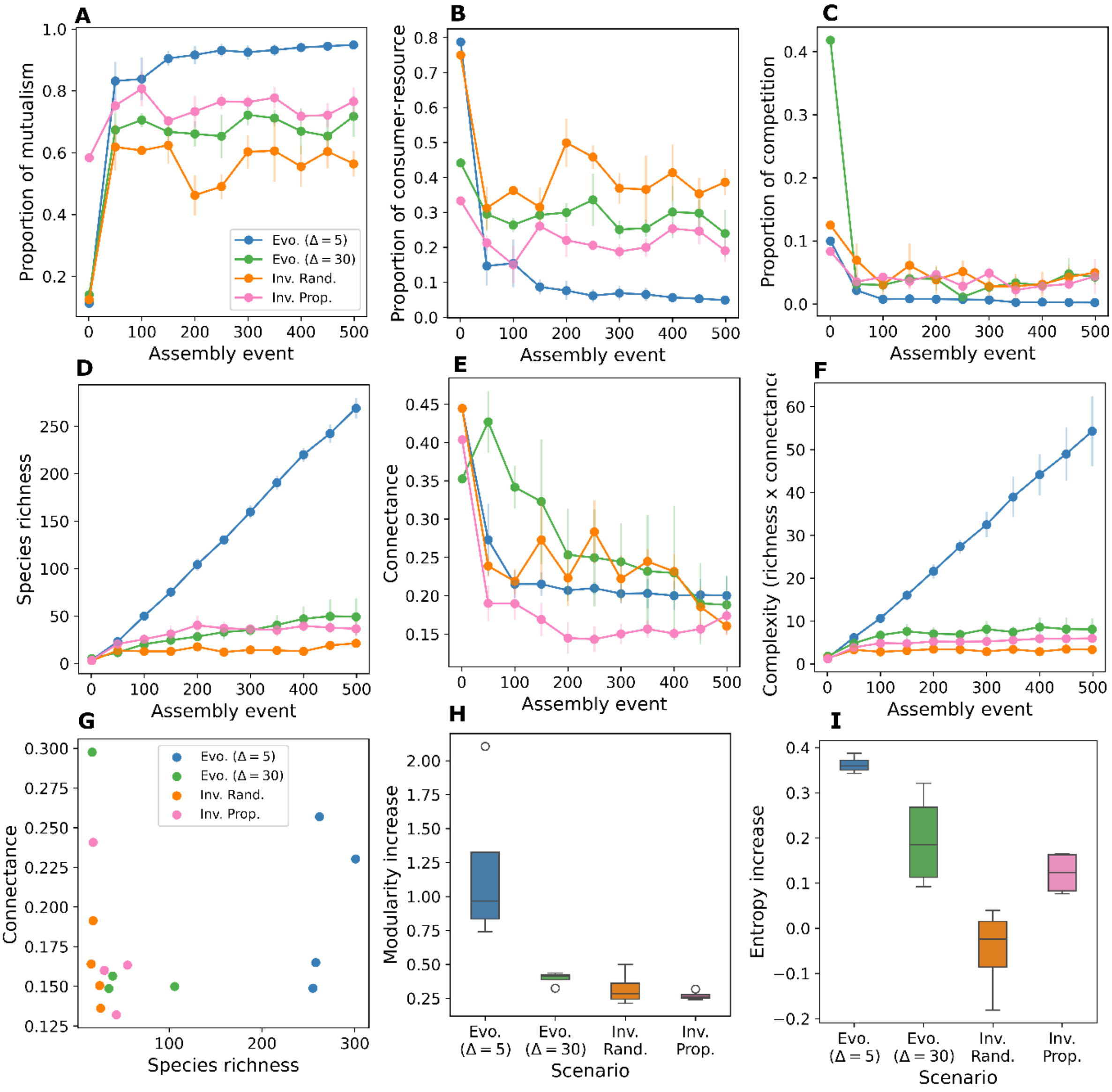

Figure S21. **Type 2 scenarios with lower intraspecific competition (mean of lognormal is -0.4 instead of -2.2).** Interestingly, lowering the intraspecific competition in Type 2 communities causes them to generally not grow to high richness, except for Evo 5 communities, which remain unchanged. A hypothesis is that, with more resources and capacity for species establishment, dominance and competitive exclusion might be enhanced. Evo 5, however, harbours more similar species, which might diminish the potential for dominance, thus prompting a partition of niches. See more details about the panels in the text above (text before Fig. S3).

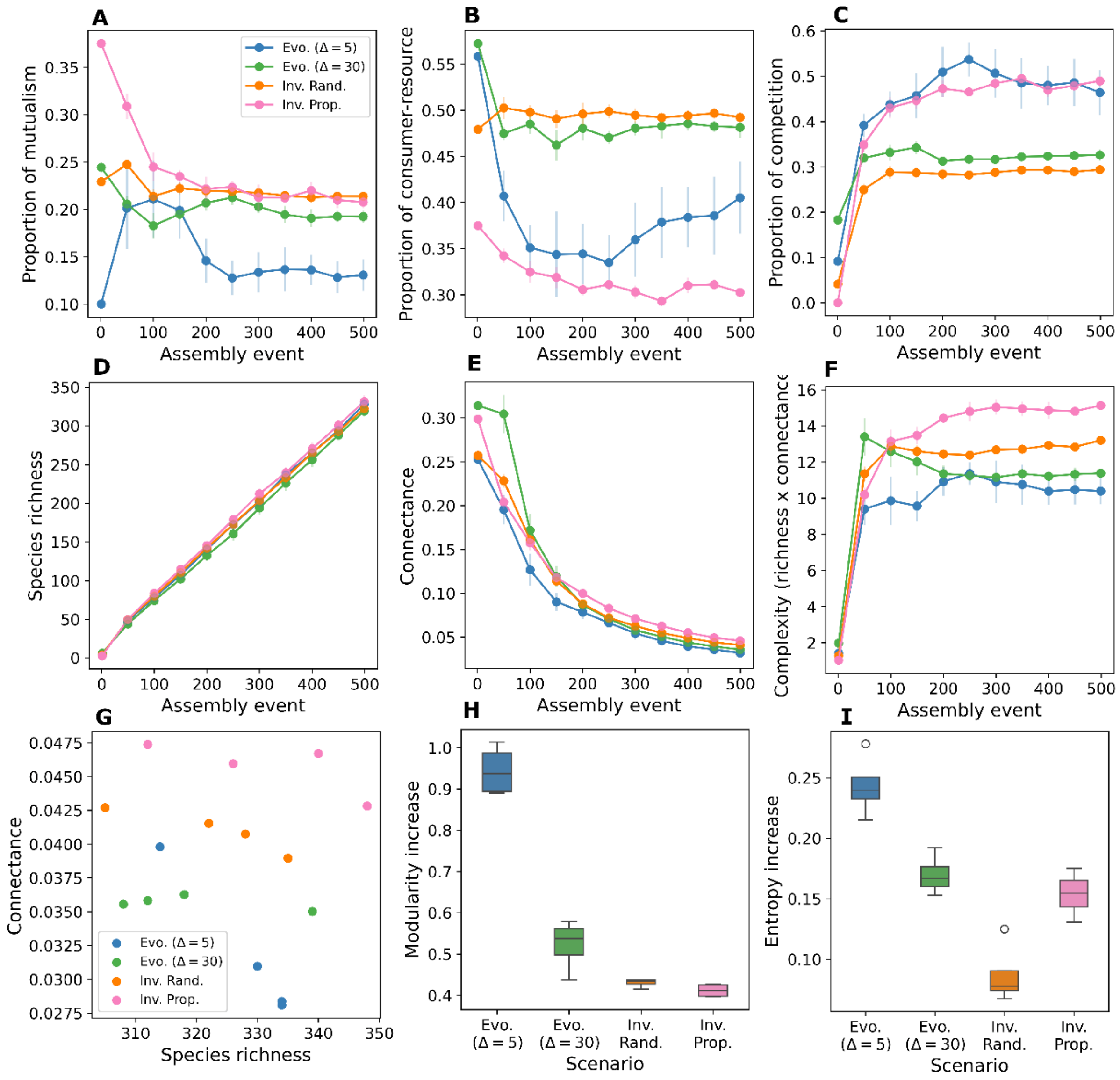

Figure S22. **Type 2 scenarios with higher intraspecific competition (mean of lognormal is -4 instead of -2.2).** As seen in Fig. S2, increasing the intraspecific competition in Type 2 communities shifts the behaviour to Type 1 communities. See more details about the panels in the text above (text before Fig. S3).

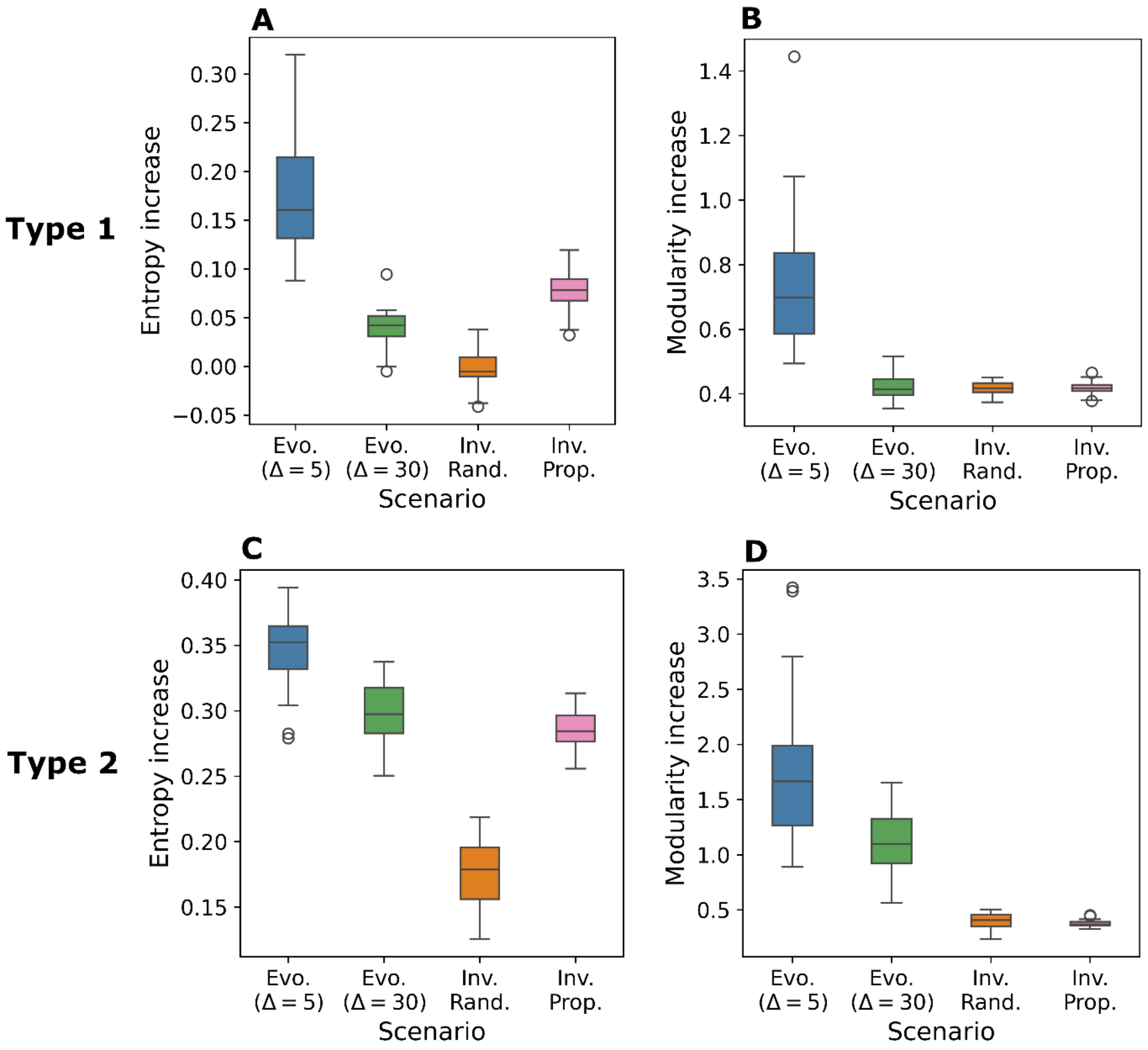

Figure S23. **Topological network metrics.** Degree entropy and modularity of n=20 simulation samples after 500 assembly events, for each scenario (details in Methods). Metrics were analysed as the relative increase from the average random network with the same complexity, calculated with n=50 samples. Values were subtracted by the random average and then divided by it, resulting in a relative increase and measuring how much is driven by the structure generated in the assembly process. (A-B) In Type 1 communities, strong inheritance is the main factor driving the increase of both entropy and modularity. (C-D) In Type 2 communities, the same holds for modularity, with weak inheritance being relevant as well, but ecological selection is also a substantial driver of entropy. Parameter values are the same as in Figures 1 and 2.

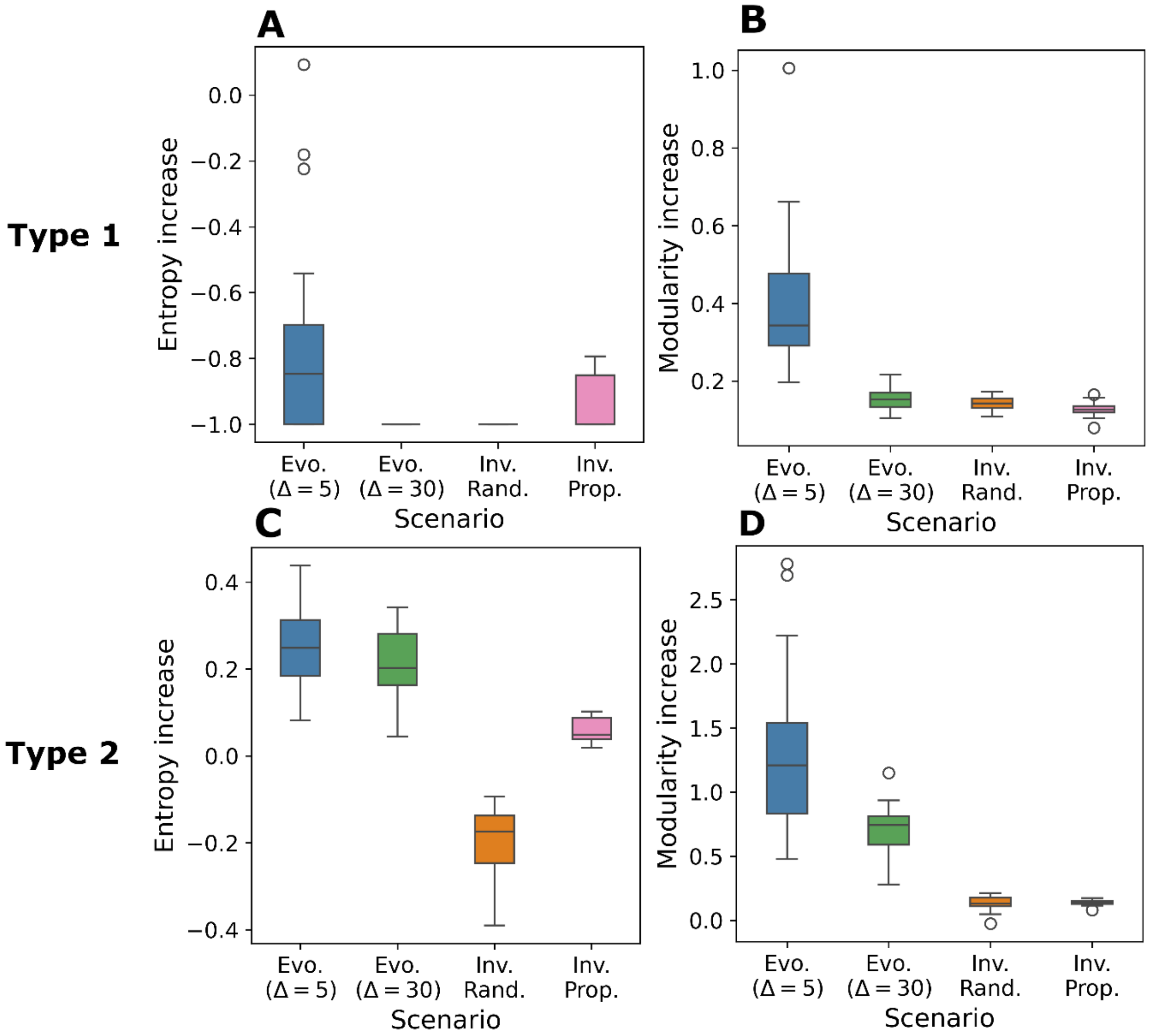

Figure S24. **Weighted network metrics.** Degree entropy and modularity calculated in the same way as in Fig S23, but considering interaction strength (apart from the saturating denominator of Type II functional responses). (A-B) In Type 1 communities, strong inheritance is still impactful, but ecological selection is also important for degree entropy. Moreover, degree entropy is now decreasing in relation to the random counterparts, much stronger than before. Weighted modularity is equivalent to the topological one. (C-D) In Type 2 communities, everything is generally the same as in the topological metrics.

Supplementary Note 2: Complementary data analysis scenarios (Figs S25 to S29)

We used empirical data from the human microbiome to evaluate how well each model scenario replicates macroecological patterns observed in microbial communities. These patterns are considered universal and are illustrated here using time-series data from gut, palm, and mouth microbiomes across multiple individuals (black dots). In all panels, the top plots show the distribution of standardised log-mean relative abundances (mean abundance distributions, MAD), with each dot representing the probability density of observing a species with a given abundance. Empirical MADs approximate a lognormal distribution, although the lower tail is truncated due to detection limits in sampling. The bottom plots show the distribution of pairwise correlation coefficients between species' relative abundances, where each dot indicates the probability density of observing a given correlation as abundances fluctuate over time. Coloured dots represent results from simulations. Figures S25 and S26 correspond to Type 1 and Type 2 communities, displaying scenarios not shown in Fig 4. Figure S27 shows the Wasserstein distance between distributions of the pairwise correlations (the second macroecological pattern shown). The plots consider all pairs between two sets of samples for each category. Empirical-Empirical corresponds to distances within data samples (all datasets together), and it shows the variations between observed distributions. Simulated-simulated shows distances between model samples, the variation within simulations. Logistic-logistic shows distances within the same simulated communities when timeseries were drawn without interactions (discarding the initial datapoints of the timeseries simulation as warm-up, for communities to adjust their equilibrium without interactions), with only logistic dynamics. The distinction between Simulated-simulated and logistic-logistic might correspond to the effects of interactions. Empirical-simulated shows distances between data samples and simulated samples, while empirical-logistic shows distances between data samples and the simulations without interactions. The distinction between these two might correspond to how much interactions help or disrupt the match between data and simulations. The closer the distributions in empirical-empirical, simulated-simulated, and empirical-simulated, in relation to logistic-logistic and empirical-logistic, the better the model with interactions is to represent the data. Mean and standard deviation of the Wasserstein metrics (Fig. S27):

	Empirical-empirical	Simulated-simulated	Logistic-logistic	Empirical-simulated	Empirical-logistic
Type 1 (Evo 5)	0.02 (±0.05)	0.0044 (±0.003)	0.001 (±0.0005)	0.071 (±0.008)	0.0625 (±0.006)
Type 2 - Evo 5	0.02 (±0.05)	0.0036 (±0.002)	0.0014 (±0.0007)	0.0459 (±0.0093)	0.0625 (±0.006)
Type 2 - Evo 30	0.02 (±0.05)	0.008 (±0.0048)	0.0019 (±0.0006)	0.038 (±0.007)	0.0624 (±0.0065)
Type 2 - Inv. Rand.	0.02 (±0.05)	0.0939 (±0.0656)	0.006 (±0.0021)	0.1147 (±0.065)	0.0625 (±0.0067)
Type 2 - Inv. Prop.	0.02 (±0.05)	0.0045 (±0.0032)	0.0017 (±0.0006)	0.038 (±0.0065)	0.0627 (±0.0065)

Figures S28 and S29 show the analysis for two of the extended scenarios from the supplementary material, each with n=20 simulated samples, for more accuracy (instead of n=4 as all above). Fig. S28 shows the mixed assembly scenario with 20% Evo. 5 and 80% Inv. Prop. (shown above in Fig. S14). Fig. S29 shows the Type 2 Evo. 30 scenario with lower intraspecific competition (shown above in Fig. S21). Both give an interesting match with the data.

Simulations were performed using the same parameter values as in the main analyses. We generated n=20 replicate communities per scenario, each assembled through 500 events. To produce time-series reflecting stochastic fluctuations around equilibrium, we added normally distributed environmental noise (standard deviation 0.1) independently to each species, scaled to its abundance.

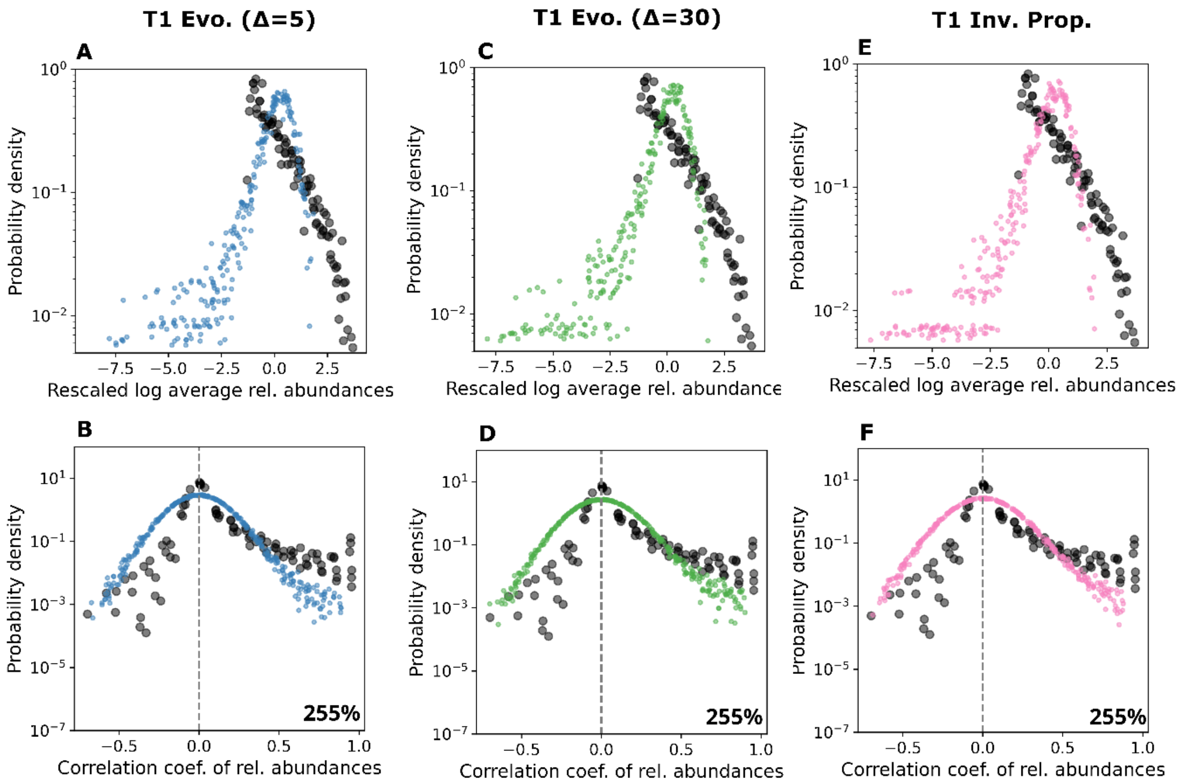

Figure S25. **Macroecological patterns for other scenarios of Type 1 communities.** (A-B) Strong inheritance. (C-D) Weak inheritance. (E-F) Invasion with variable interaction-type proportions. See more details in the text above.

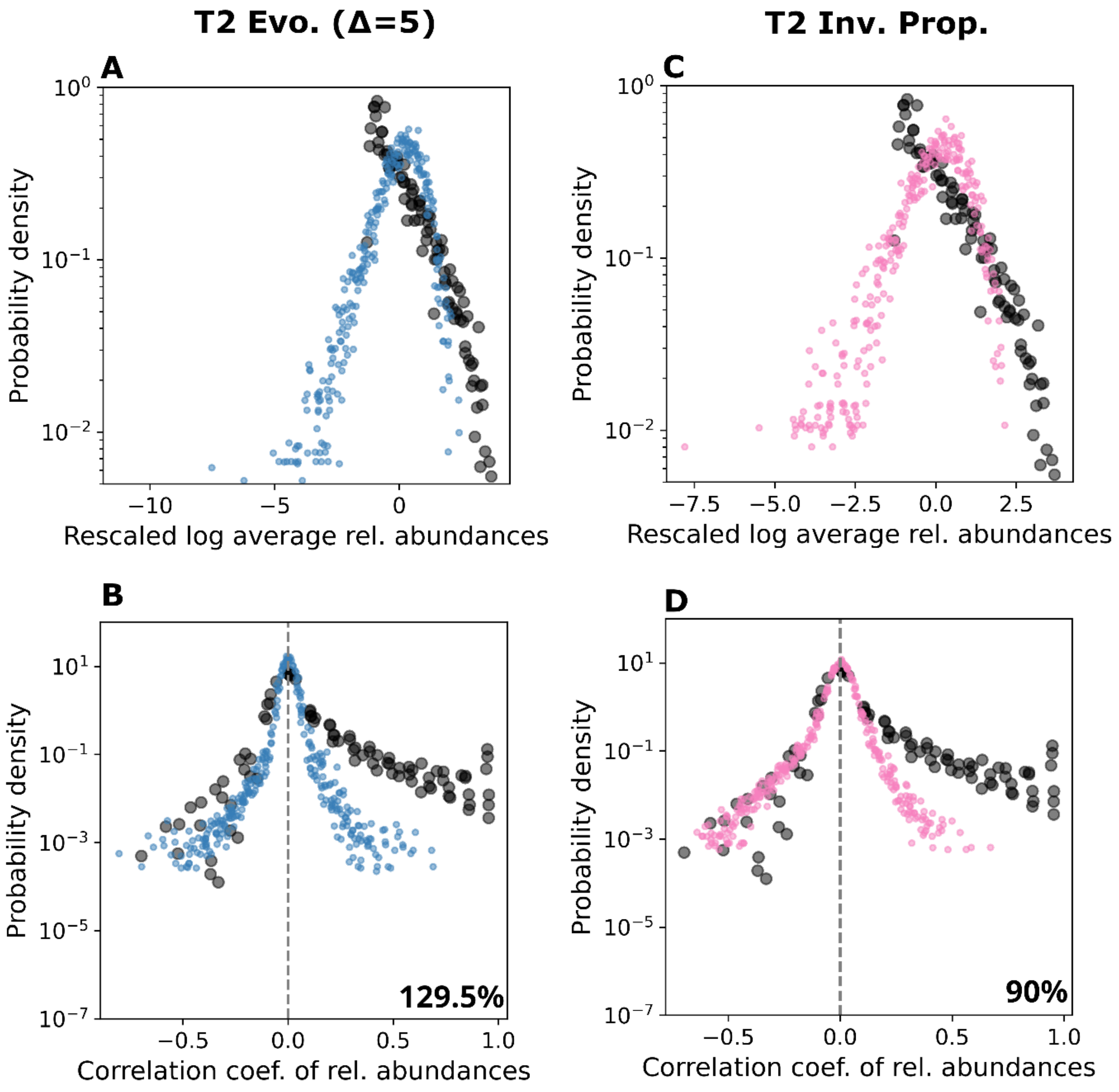

Figure S26. **Macroecological patterns for other scenarios of Type 2 communities.** (A-B) Strong inheritance. (C-D) Invasion with variable interaction-type proportions. See more details in the text above.

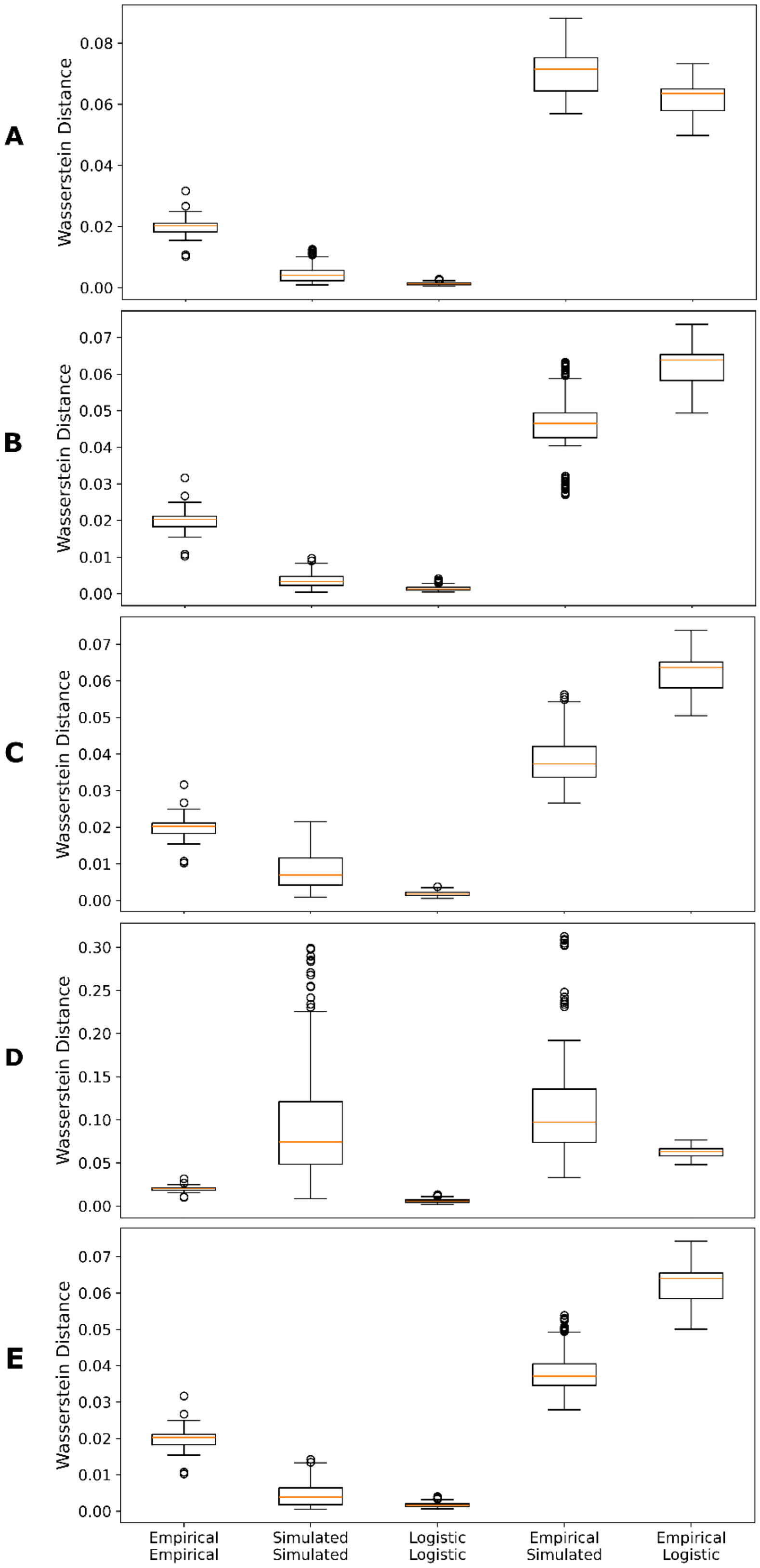

Figure S27. **Wasserstein distances between pairwise correlations.** (A) Type 1 communities (all generally the same), (B) Type 2 Evo. 5, (C) Type 2 Evo. 30, (D) Type 2 Inv. Rand., (E) Type 2 Inv. Prop.. Type 2 better represents the data, possibly Evo 30. See more details in the text above.

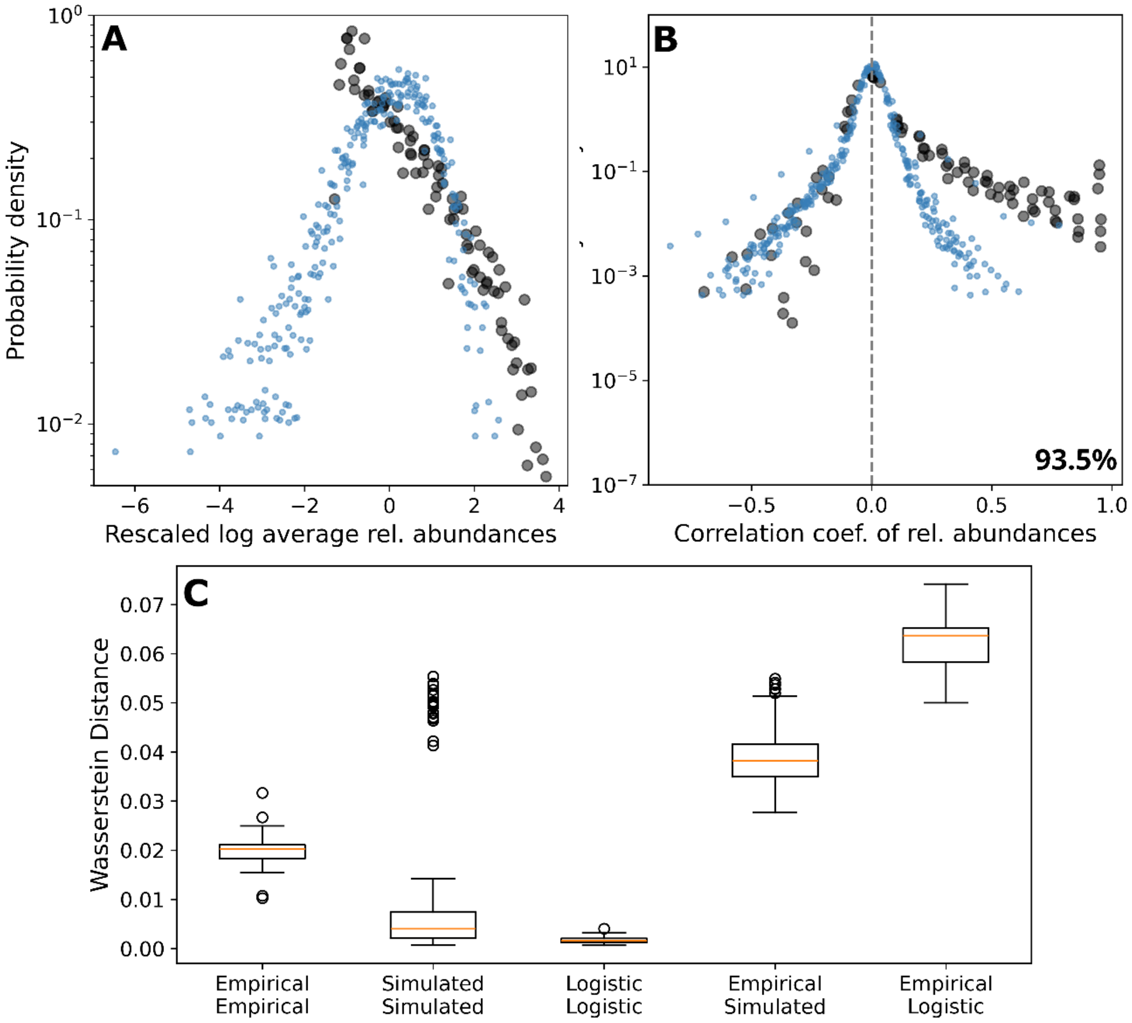

Figure S28. **Macroecological patterns and Wasserstein distance for Type 2 with mixed evolution and invasion.** Simulations of 20% Evo. 5 and 80% Inv. Prop. assembly events, n=20 samples. (A) Mean abundance distributions (MAD), (B) pairwise correlation distributions, (C) Wasserstein distance for correlation distributions. See more details in the text above.

(C) Mean and standard deviation:

Empirical-empirical	Simulated-simulated	Logistic-logistic	Empirical-simulated	Empirical-logistic
0.02 (±0.05)	0.0089 (±0.0138)	0.0017 (±0.0006)	0.0387 (±0.0069)	0.0626 (±0.0065)

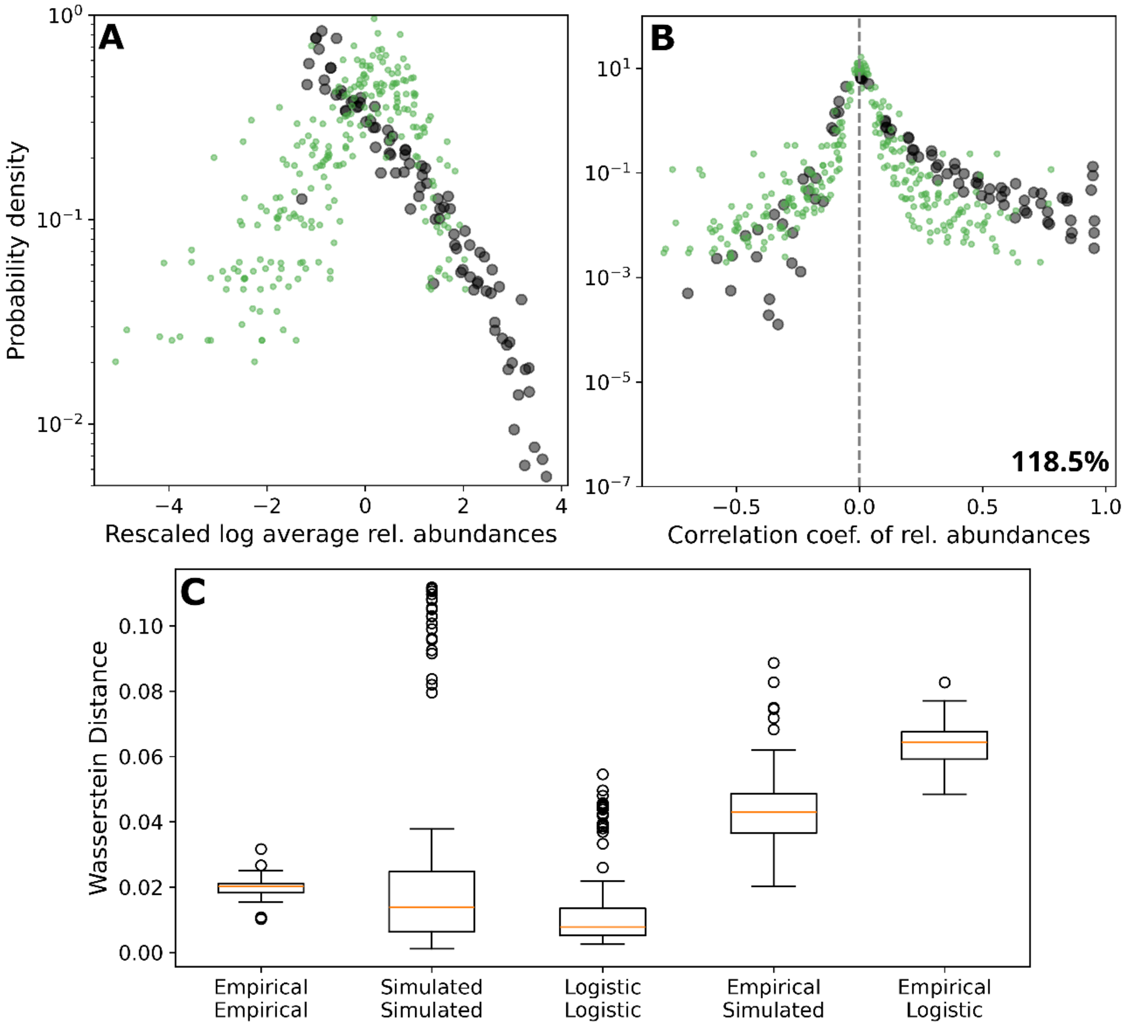

Figure S29. **Macroecological patterns and Wasserstein distance for Type 2 with lower intraspecific competition (mean of lognormal is -0.4 instead of -2.2).** Simulations of Evo. 30, n=20 samples. (A) Mean abundance distributions (MAD), (B) pairwise correlation distributions, (C) Wasserstein distance for correlation distributions. See more details in the text above.

(C) Mean and standard deviation:

Empirical-empirical	Simulated-simulated	Logistic-logistic	Empirical-simulated	Empirical-logistic
0.02 (±0.05)	0.0226 (±0.0274)	0.0119 (±0.0113)	0.0437 (±0.0117)	0.0635 (±0.0071)
