# Supplementary material for: The eco-evolutionary assembly of complex communities with multiple interaction types
Source: Nat Commun. 2026 Mar 5;17:3511. doi: 10.1038/s41467-026-70117-8 (PMC13083869; doi:10.1038/s41467-026-70117-8)
Supplement: Supplementary file 2 — Reporting Summary [file 41467_2026_70117_MOESM2_ESM.pdf]

nature portfolio

Corresponding author(s): Miguel Lurgi (miguel.lurgi@swansea.ac.uk)

Last updated by author(s): 13/02/2026

Reporting Summary

Nature Portfolio wishes to improve the reproducibility of the work that we publish. This form provides structure for consistency and transparency in reporting. For further information on Nature Portfolio policies, see our Editorial Policies and the Editorial Policy Checklist.

Statistics

For all statistical analyses, confirm that the following items are present in the figure legend, table legend, main text, or Methods section.

n/a	Confirmed	
☐	☒	The exact sample size (*n*) for each experimental group/condition, given as a discrete number and unit of measurement
☒	☐	A statement on whether measurements were taken from distinct samples or whether the same sample was measured repeatedly
☒	☐	The statistical test(s) used AND whether they are one- or two-sided<br>*Only common tests should be described solely by name; describe more complex techniques in the Methods section.*
☒	☐	A description of all covariates tested
☒	☐	A description of any assumptions or corrections, such as tests of normality and adjustment for multiple comparisons
☐	☒	A full description of the statistical parameters including central tendency (e.g. means) or other basic estimates (e.g. regression coefficient) AND variation (e.g. standard deviation) or associated estimates of uncertainty (e.g. confidence intervals)
☒	☐	For null hypothesis testing, the test statistic (e.g. *F*, *t*, *r*) with confidence intervals, effect sizes, degrees of freedom and *P* value noted<br>*Give P values as exact values whenever suitable.*
☒	☐	For Bayesian analysis, information on the choice of priors and Markov chain Monte Carlo settings
☒	☐	For hierarchical and complex designs, identification of the appropriate level for tests and full reporting of outcomes
☒	☐	Estimates of effect sizes (e.g. Cohen's *d*, Pearson's *r*), indicating how they were calculated

Our web collection on statistics for biologists contains articles on many of the points above.

Software and code

Policy information about availability of computer code

Data collection	no data was collected
Data analysis	Files generated and analysed in this study and computer code developed to implement the model and execute the numerical simulations are available in the accompanying Zenodo repository [https://doi.org/10.5281/zenodo.15621027]. Also on GitHub [https://github.com/computational-ecology-lab/eco-evo-network-assembly].<br>All simulations and data analyses were performed in Python (v3.11.11). The computational workflow relied on NumPy (v2.2.6) and SciPy (v1.15.2) for numerical computations, pandas (v2.2.3) for data handling, and Matplotlib (v3.9.1) and seaborn (v0.13.2) for visualisation. Additional specialised visualisations were generated using python-ternary (v1.0.8).

For manuscripts utilizing custom algorithms or software that are central to the research but not yet described in published literature, software must be made available to editors and reviewers. We strongly encourage code deposition in a community repository (e.g. GitHub). See the Nature Portfolio guidelines for submitting code & software for further information.

Data

Policy information about availability of data

All manuscripts must include a data availability statement. This statement should provide the following information, where applicable:

- Accession codes, unique identifiers, or web links for publicly available datasets
- A description of any restrictions on data availability
- For clinical datasets or third party data, please ensure that the statement adheres to our policy

No new experimental or field data were collected. Publicly available datasets from the EBI MGnify platform were used for model comparison, specifically longitudinal human microbiome data from subjects F4 and M3 in MGYS00002184 [https://www.ebi.ac.uk/metagenomics/projects/MGYS00002184].
Raw OTU data were filtered and transformed as described in the Methods, and the original tables and process are available in the Zenodo repository [https://doi.org/10.5281/zenodo.15621027].

Research involving human participants, their data, or biological material

Policy information about studies with human participants or human data. See also policy information about sex, gender (identity/presentation), and sexual orientation and race, ethnicity and racism.

Reporting on sex and gender	n/a
Reporting on race, ethnicity, or other socially relevant groupings	n/a
Population characteristics	n/a
Recruitment	n/a
Ethics oversight	n/a

Note that full information on the approval of the study protocol must also be provided in the manuscript.

Field-specific reporting

Please select the one below that is the best fit for your research. If you are not sure, read the appropriate sections before making your selection.

☐ Life sciences ☐ Behavioural & social sciences ☒ Ecological, evolutionary & environmental sciences

For a reference copy of the document with all sections, see nature.com/documents/nr-reporting-summary-flat.pdf

Ecological, evolutionary & environmental sciences study design

All studies must disclose on these points even when the disclosure is negative.

Study description	This is a theoretical study that analyses a model of eco-evolutionary community assembly with multiple interaction types, exploring several distinct scenarios. We performed computational simulations and used publicly available data to compare our model results with empirical macroecological patterns found in microbial communities. No experiments were performed and no data was empirically collected.
Research sample	The dataset consists of longitudinal human microbiome samples from two healthy adult individuals (one female, one male) collected at four body sites over 396 timepoints. No experimental manipulations were performed. The data provide dense timeseries measurements of microbial taxa, allowing the study of temporal dynamics and individual-specific fluctuations in microbiota. The dataset was selected to evaluate model performance on high-resolution longitudinal human microbiome data. Data source: MGYS00002184 [https://www.ebi.ac.uk/metagenomics/studies/MGYS00002184#overview].
Sampling strategy	n/a
Data collection	n/a
Timing and spatial scale	n/a
Data exclusions	n/a
Reproducibility	n/a
Randomization	n/a
Blinding	n/a

Did the study involve field work? ☐ Yes ☒ No

Reporting for specific materials, systems and methods

We require information from authors about some types of materials, experimental systems and methods used in many studies. Here, indicate whether each material, system or method listed is relevant to your study. If you are not sure if a list item applies to your research, read the appropriate section before selecting a response.

Materials & experimental systems

n/a	Involved in the study	
☒	☐	Antibodies
☒	☐	Eukaryotic cell lines
☒	☐	Palaeontology and archaeology
☒	☐	Animals and other organisms
☒	☐	Clinical data
☒	☐	Dual use research of concern
☒	☐	Plants

Methods

n/a	Involved in the study	
☒	☐	ChIP-seq
☒	☐	Flow cytometry
☒	☐	MRI-based neuroimaging

Plants

Seed stocks	n/a
Novel plant genotypes	n/a
Authentication	n/a
